# Diltiazem inhibits SARS-CoV-2 cell attachment and internalization and decreases the viral infection in mouse lung

**DOI:** 10.1371/journal.ppat.1010343

**Published:** 2022-02-17

**Authors:** Xinxin Wang, Jie Luo, Zhiyuan Wen, Lei Shuai, Chong Wang, Gongxun Zhong, Xijun He, Huizhen Cao, Renqiang Liu, Jinying Ge, Ronghong Hua, Ziruo Sun, Xijun Wang, Jinliang Wang, Zhigao Bu

**Affiliations:** 1 State Key Laboratory of Veterinary Biotechnology, Harbin Veterinary Research Institute, Chinese Academy of Agricultural Sciences, Harbin, People’s Republic of China; 2 Jiangsu Co-innovation Center for Prevention and Control of Important Animal Infectious Diseases and Zoonoses, Yangzhou University, Yangzhou, P. R. China; University of Iowa, UNITED STATES

## Abstract

The continuous emergence of severe acute respiratory coronavirus 2 (SARS-CoV-2) variants and the increasing number of breakthrough infection cases among vaccinated people support the urgent need for research and development of antiviral drugs. Viral entry is an intriguing target for antiviral drug development. We found that diltiazem, a blocker of the L-type calcium channel Ca_v_1.2 pore-forming subunit (Ca_v_1.2 α_1c_) and an FDA-approved drug, inhibits the binding and internalization of SARS-CoV-2, and decreases SARS-CoV-2 infection in cells and mouse lung. Ca_v_1.2 α_1c_ interacts with SARS-CoV-2 spike protein and ACE2, and affects the attachment and internalization of SARS-CoV-2. Our finding suggests that diltiazem has potential as a drug against SARS-CoV-2 infection and that Ca_v_1.2 α_1c_ is a promising target for antiviral drug development for COVID-19.

## Author summary

The emergence of variants of SARS-CoV-2 and the breakthrough infections that have occurred in recipients of approved SARS-CoV-2 vaccines raise doubts about the effectiveness of the vaccines and highlight the importance of antiviral drugs. An ideal drug to treat COVID-19 should be safe, affordable, and accessible. However, remdesivir remains the only authorized drug approved by the US FDA for emergency use, and it appears to have little effect on hospitalized COVID-19 patients. Therefore, identifying drugs to treat SARS-CoV-2 infections remains extremely important and urgent. In this study, we found that the calcium channel blocker diltiazem, which has been approved in the US since 1982 and is cheap and widely used in clinical practice for many indications, inhibits the binding and internalization of SARS-CoV-2, and decreases SARS-CoV-2 infection in cells and mouse lung. The L-type calcium channel Ca_v_1.2 pore-forming subunit (Ca_v_1.2 α_1c_), the main target of diltiazem, interacts and colocalizes with SARS-CoV-2 spike protein and ACE2, thereby affecting cell attachment and internalization of SARS-CoV-2. Our finding suggests that diltiazem could be candidate COVID-19 treatment and that Ca_v_1.2 α_1c_ may be a promising target for anti-SARS-CoV-2 drugs.

## Introduction

As of December 29, 2021, the COVID-19 pandemic has resulted in more than 280 million confirmed cases and approximately 5.4 million deaths worldwide according to the World Health Organization. A novel coronavirus, named severe acute respiratory syndrome coronavirus 2 (SARS-CoV-2), was identified as the causative pathogen of the disease. SARS-CoV-2 is an enveloped non-segmented positive RNA virus that belongs to the betacoronavirus genus [1]. The spike (S) protein of SARS-CoV-2 is responsible for binding to cellular receptors and subsequent viral entry into host cells. SARS-CoV-2 S protein is composed of the S1 and S2 subdomains. S1 contains the receptor-binding domain (RBD) and is responsible for binding to specific receptors. S2 contains a fusion peptide and is responsible for the fusion of the viral membrane with the cellular membrane [2,3]. Angiotensin-converting enzyme 2 (ACE2) is a recognized cellular receptor for SARS-CoV-2 [1,4,5]. Recent studies have revealed other potential receptors and entry factors [4,6–17]. After binding to the cell surface, SARS-CoV-2 enters cells via receptor-mediated endocytosis [18,19] and transmembrane serine protease 2 (TMPRSS2)-mediated direct fusion with the plasma membrane [4].

The continuous emergence of SARS-CoV-2 variants and the increasing number of breakthrough infection cases among vaccinated people suggest that antiviral drugs that are effective against SARS-CoV-2 are urgently needed. Viral entry is the early stage of infection and an intriguing target for antiviral drug development. Exploring the underlying mechanisms of receptor recognition, cell binding, and internalization are essential to the design of antiviral drugs to block early infection.

Previous studies have revealed that calcium (Ca^2+^) and calcium channels are important for the infection of numerous viruses [20], including severe acute respiratory syndrome coronavirus (SARS-CoV) [21] and Middle East respiratory syndrome coronavirus (MERS-CoV) [22]. There are many different plasma-membrane calcium channels [23], including the voltage-dependent calcium channels (VDCCs), which are the best characterized. VDCCs are classified into three different families: (i) L-type calcium channels; (ii) P/Q-type, N-type, and R-type calcium channels; and (iii) T-type calcium channels [23]. VDCCs have been reported to affect the cell binding and internalization of several viruses [24–27]. For example, calcium voltage-gated channel subunit alpha1 S mediates New World arenavirus binding and internalization, and benidipine hydrochloride, an inhibitor of L-type calcium channels, inhibits the severe fever associated with thrombocytopenia syndrome virus infection by interfering with virus internalization and reducing viral replication. Whether VDCCs are important for SARS-CoV-2 infection remains unclear. In this study, we found that the FDA-approved drug diltiazem, a blocker of the L-type calcium channel Ca_v_1.2 pore-forming subunit (Ca_v_1.2 α_1c_), decreases the cell binding and internalization of SARS-CoV-2 and inhibits SARS-CoV-2 infection in cells and mice. Our finding suggests that diltiazem has potential as a treatment for SARS-CoV-2 infection and that Ca_v_1.2 α_1c_ is a promising target for antiviral drug development for COVID-19.

## Results

### Diltiazem inhibits SARS-CoV-2 infection in cells

**Fig 1:**
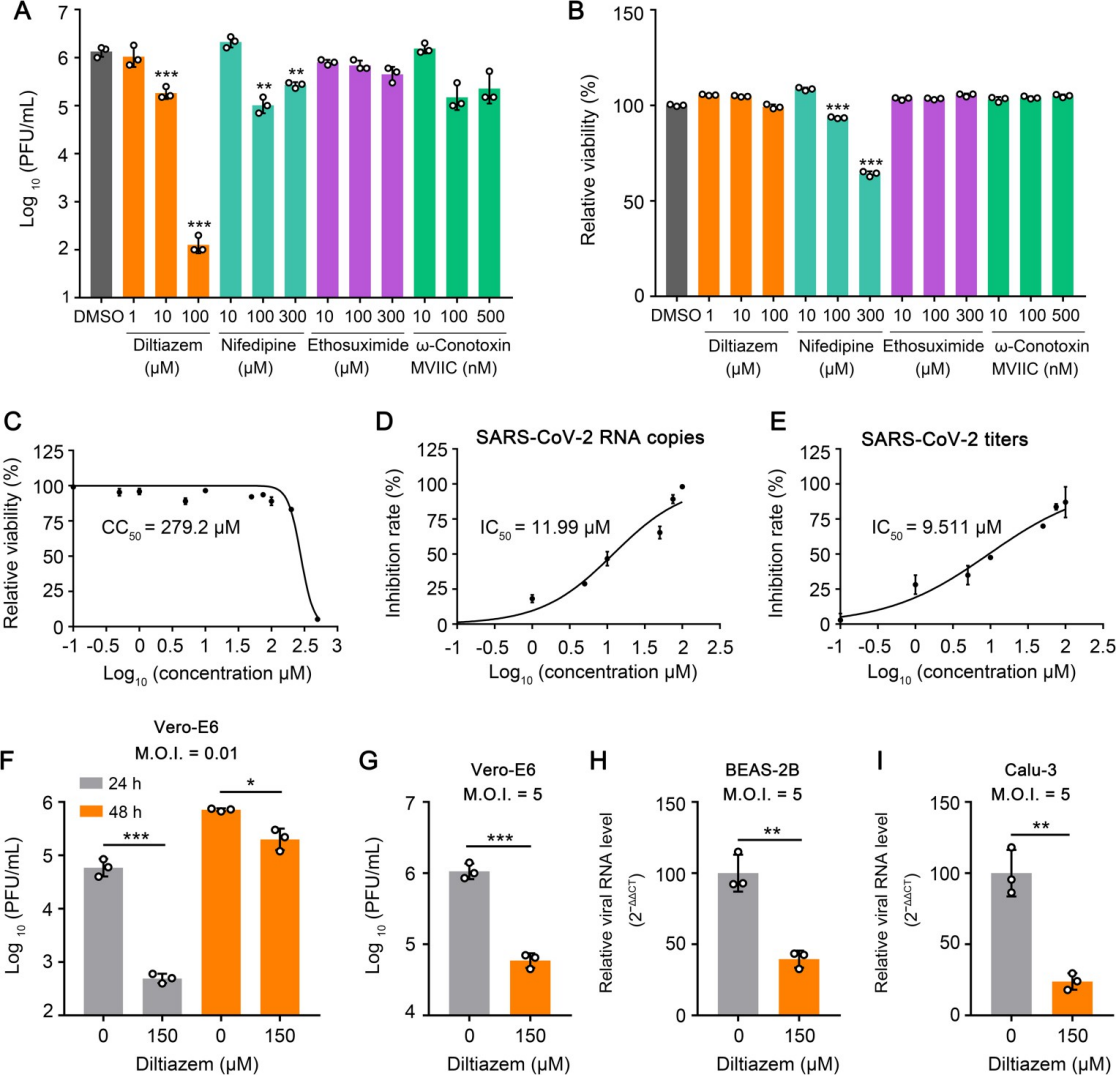
Diltiazem inhibits SARS-CoV-2 infection in cells. (A) Vero-E6 cells were treated with DMSO or inhibitors at the indicated concentrations for 1 h, and then infected with HRB25 (M.O.I. = 0.01). The supernatants were harvested at 24 h post-infection for plaque assays. PFU, plaque-forming units. (B) The viability of Vero-E6 cells was determined in the presence of inhibitors at the indicated concentrations. (C) Vero-E6 cells were treated with diltiazem at different concentrations or vehicle to determine the CC_50_. CC_50_: 50% cytotoxic concentration. (D and E) IC_50_ of diltiazem to HRB25 infectivity. Vero-E6 cells were treated with vehicle or diltiazem at the indicated concentrations for 1 h, and then infected with HRB25 (M.O.I. = 0.01); the supernatants were harvested at 24 h post-infection to determine viral RNA copy numbers (D) and virus titers (E) by use of qPCR and plaque assays, respectively. IC_50_: 50% inhibitory concentration. (F and G) Vero-E6 cells were treated with diltiazem for 1 h, and then infected with HRB25 at an M.O.I. of 0.01 (F) or 5 (G); the supernatants were harvested at the indicated timepoints for plaque assays. (H and I) BEAS-2B cells (H) and Calu-3 cells (I) were treated with diltiazem for 1 h, and then infected with HRB25 (M.O.I. = 5). At 24 h post-infection, the viral RNA level in the cell lysate was measured by qPCR. The data shown are the means ± SDs of three independent experiments or replicates. The two-tailed unpaired Student’s t-test was used for the statistical analysis. *p < 0.05, **p < 0.01, ***p < 0.001.

It has been reported that VDCCs affect the cell binding and internalization of several viruses [24–27]. To test whether VDCCs are required for SARS-CoV-2 infection, we chose four inhibitors of different channels (diltiazem and nifedipine, which inhibit L-type calcium channels [28]; ω-Conotoxin MVIIC, which inhibits P/Q- and N-type calcium channels [29]; and ethosuximide, which inhibits T-type calcium channels [30,31]) to perform a screening assay in Vero-E6 cells for potential drugs against SARS-CoV-2 infection. Vero-E6 cells were treated with the drugs at different concentrations for 1 h at 37°C, and then infected with a SARS-CoV-2 human isolate (HRB25) [32] at a multiplicity of infection (M.O.I.) of 0.01. The infectious titers in the supernatants of the infected cells were evaluated at 24 h post-infection. Diltiazem showed low toxicity and significantly inhibited HRB25 infection of Vero-E6 cells (Fig 1A and 1B). The 50% cytotoxic concentration (CC_50_) of diltiazem in Vero-E6 cells was 279.2 μΜ (Fig 1C). The 50% maximal inhibitory concentration (IC_50_) of diltiazem for HRB25 infection of Vero-E6 cells was 11.99 μΜ and 9.511 μΜ based on the RNA copies and virus titers, respectively (Fig 1D and 1E). Diltiazem-mediated inhibition of SARS-CoV-2 infection was also detected at 48 h post-infection (Fig 1F). We then infected diltiazem-treated Vero-E6 cells at an M.O.I. of 5, and confirmed that diltiazem inhibited HRB25 infection of Vero-E6 cells at a high infection dose (Fig 1G). We further tested whether diltiazem inhibits SARS-CoV-2 infection of human airway epithelial cells (BEAS-2B cells and Calu-3 cells) by qPCR at 24 h post-infection. The results showed that diltiazem significantly inhibits HRB25 infection of both cell types (Fig 1H and 1I).

### Diltiazem affects the early stage of SARS-CoV-2 infection

**Fig 2:**
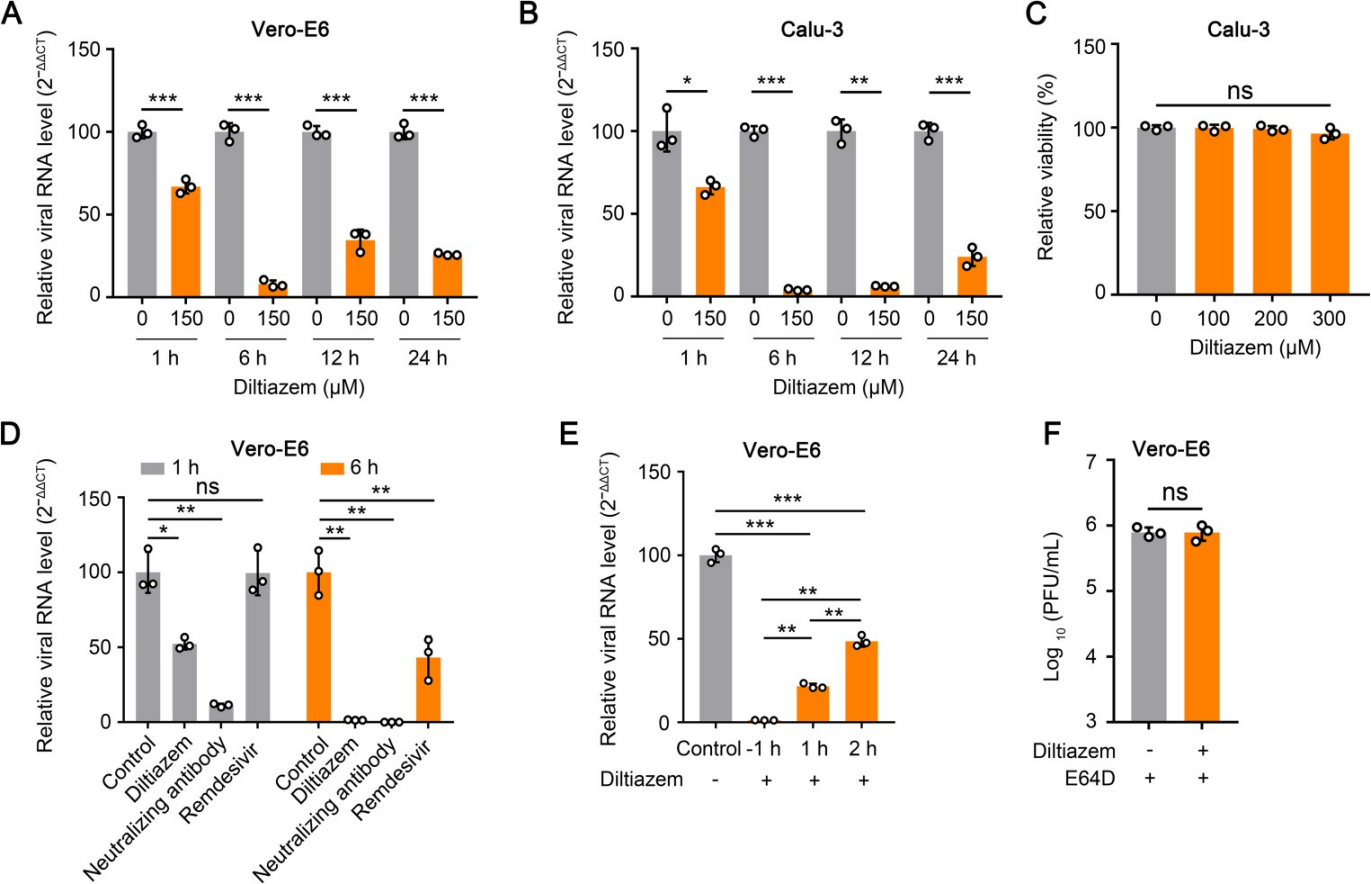
Diltiazem inhibits the early stage of SARS-CoV-2 infection *in vitro*. (A and B) Vero-E6 cells (A) and Calu-3 cells (B) were incubated with vehicle or diltiazem for 1 h, and then infected with HRB25 at a M.O.I. of 5 and a M.O.I. of 10, respectively. At the indicated timepoints post-infection, the viral RNA level in the cell lysates were measured by qPCR. (C) The viability of Calu-3 cells was determined in the presence of diltiazem at the indicated concentrations. (D) Vero-E6 cells were treated with diltiazem and remdesivir for 1 h, respectively, and then infected with HRB25 (M.O.I. = 5). For the virus neutralization assay, HRB25 (M.O.I. = 5) was incubated with neutralizing antibody (20 μg/ml) for 1 h at 4°C, and then the mixture was used to infect Vero-E6 cells. At the indicated timepoints post-infection, the viral RNA level in the cell lysate was measured by qPCR. (E) Vero-E6 cells were infected with HRB25 (M.O.I. = 5), and diltiazem was added at -1 h, 1 h, or 2 h post-infection. The viral RNA level in the cell lysate was determined at 6 h post-infection by qPCR. (F) Vero-E6 cells were infected with HRB25 (M.O.I. = 5), then diltiazem and E64D were added at 6 h post-infection. The supernatants were harvested at 24 h post-infection for plaque assays. The data shown are the means ± SDs of three independent experiments or replicates. The two-tailed unpaired Student’s t-test was used for the statistical analysis. ns, not significant, *p < 0.05, **p < 0.01, ***p < 0.001.

We next determined which stage of SARS-CoV-2 infection was affected by diltiazem. Vero-E6 cells were treated with 150 μΜ diltiazem for 1 h, and then infected with HRB25 (M.O.I. = 5). The viral RNA level in the cell lysate relative to that of β-actin was determined by qPCR at 1 h, 6 h, 12 h, and 24 h post-infection. The results showed that compared with control cells (0 μΜ), diltiazem treatment significantly decreased the viral RNA level as early as 1 h post-infection, indicating that diltiazem inhibits the early stage of SARS-CoV-2 infection (Fig 2A). We confirmed these result in Calu-3 cells by using qPCR to detect the viral RNA level in the cell lysate relative to the 28S rRNA level and found that diltiazem treatment significantly decreased the viral RNA level as early as 1 h post-infection (Fig 2B and 2C).

We then used a neutralizing antibody against the SARS-CoV-2 S protein [33] and remdesivir, a well-known inhibitor of SARS-CoV-2 replication, to perform parallel tests as described above in Vero-E6 cells [34], and detected the viral RNA level in the cell lysate relative to the β-actin level at 1 h and 6 h post-infection. The results showed that neutralizing antibody or diltiazem treatment significantly decreased the viral RNA level at 1 h and 6 h post-infection, whereas remdesivir treatment only decreases the viral RNA level at 6 h post-infection (Fig 2D), which indicates that diltiazem inhibits the early step of SARS-CoV-2 infection.

We also performed a time-of-addition assay to test whether diltiazem inhibits the early step of SARS-CoV-2 infection in Vero-E6 cells. Cells were treated with diltiazem at -1 h, 1 h, and 2 h after HRB25 infection (M.O.I. = 5). The viral RNA level in the cell lysate relative to the β-actin level was assessed by using qPCR at 6 h post-infection. We found that compared with the viral RNA level of control cells, adding diltiazem at -1 h, 1 h, or 2 h post-infection significantly decreased the viral RNA level, although the extent of the decrease for the 2 h post-infection timepoint was less than that for the -1 h and 1 h post-infection timepoints (Fig 2E). These results confirm that diltiazem affects the early stage of SARS-CoV-2 infection. To test whether diltiazem inhibits SARS-CoV-2 infection at a late stage in Vero-E6 cells, we added diltiazem/E64D at 6 h post-infection, and determined the viral titer at 24 h post-infection. E64D inhibits cathepsin L [35] and prevents the infection of progeny viruses. The results showed that the viral titer in diltiazem-treated Vero-E6 cells was comparable to that of control cells, indicating that diltiazem has no effect on SARS-CoV-2 infection at a late stage (Fig 2F).

### Diltiazem inhibits SARS-CoV-2 binding

**Fig 3:**
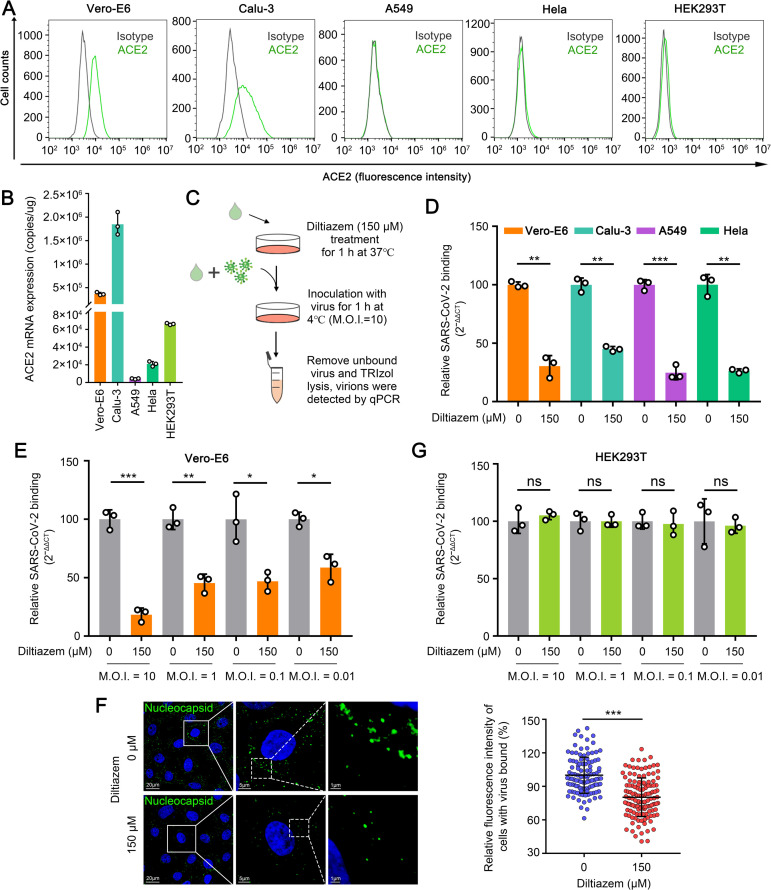
Diltiazem inhibits SARS-CoV-2 binding. (A) Cells were subjected to flow cytometry to detect the surface expression of ACE2. (B) Cells were lysed to determine ACE2 mRNA copy numbers by qPCR. (C) Schematic of the viral binding assay. (D) Diltiazem preincubated cells were incubated with HRB25 (M.O.I. = 10) at 4°C for 1 h. The viral RNA level in the cell lysate was measured by qPCR. (E) Diltiazem preincubated Vero-E6 cells were incubated with HRB25 at an M.O.I. of 10, 1, 0.1, or 0.01. The viral RNA level in the cell lysate was measured by qPCR. (F) Vero-E6 cells were treated and infected as described in (C). The cells were incubated with an anti-SARS-CoV-2 nucleocapsid rabbit monoclonal antibody, and visualized with Alexa Fluor 488-conjugated goat anti-rabbit IgG (green). Cell nuclei were stained with Hoechst 33342. Representative images are shown. The fluorescence intensities of cell-bound HRB25 in at least 110 cells per sample were quantified. (G) HEK293T cells were treated and assessed as described in (E). The data shown represent, or are from, three independent experiments or replicates (means ± SDs). The two-tailed unpaired Student’s t-test was used for the statistical analysis. ns, not significant, *p < 0.05, **p < 0.01, ***p < 0.001.

Binding to the cell surface through the S protein is the first step of the early stage of SARS-CoV-2 infection. To determine whether diltiazem inhibits SARS-CoV-2 binding, we performed viral binding assays in Vero-E6 cells or Calu-3 cells that express ACE2 at high levels, and Hela cells, A549 cells or HEK293T cells that express ACE2 at lower levels (Fig 3A and 3B). Diltiazem-treated cells were incubated with HRB25 (M.O.I. = 10) at 4°C for 1 h. The infected cells were washed with chilled PBS immediately to remove unbound viruses, and then the viral RNA level in the cell lysate relative to that of β-actin (Vero-E6 cells) or 28S rRNA (Calu-3 cells, Hela cells, and A549 cells) was determined by qPCR (Fig 3C). We found that compared with the viral RNA level of control cells, the viral RNA level of diltiazem-treated cells was significantly decreased in Vero-E6 cells, Calu-3 cells, A549 cells, and Hela cells, indicating that diltiazem inhibits SARS-CoV-2 binding to cells (Fig 3D). To exclude the possibility that the viral infection dose had an effect, we next performed viral binding assays with HRB25 at an M.O.I. of 10, 1, 0.1, or 0.01 in Vero-E6 cells as described above. Compared with the virus RNA level of the control cells, the viral RNA level of the diltiazem-treated cells was significantly decreased at all tested M.O.I.s in Vero-E6 cells, indicating that the diltiazem inhibition of SARS-CoV-2 binding is independent of the viral infection dose (Fig 3E).

To further verify the above results, we performed a microscopy-based assay to observe the SARS-CoV-2 viral particles on the cell surface. Vero-E6 cells were processed as described above. The infected cells were then fixed for immunofluorescence with an antibody against the SARS-CoV-2 nucleocapsid protein under permeabilized conditions, and stained to visualize the viral particles. The fluorescence intensity of each cell was calculated. We found that the fluorescence intensity of SARS-CoV-2 in diltiazem-treated Vero-E6 cells was significantly lower than that of the control cells (Fig 3F). These data demonstrate that diltiazem inhibits SARS-CoV-2 binding to Vero-E6 cells, Calu-3 cells, A549 cells, and Hela cells. Of note, we found that the viral RNA level in diltiazem-treated cells was comparable to that of control cells at all tested M.O.I.s in HEK293T cells (Fig 3G), indicating that diltiazem inhibits SARS-CoV-2 binding in a cell type-dependent manner.

### Diltiazem decreases the cell surface expression of ACE2

Since diltiazem is a calcium channel blocker, it could disrupt intracellular Ca^2+^ levels, blocking normal cellular functions, and it that way decrease SARS-CoV-2 binding. To test whether diltiazem inhibits SARS-CoV-2 binding by disrupt intracellular Ca^2+^ levels, we added BAPTA-AM, an intracellular calcium chelator, to our viral binding assays in Vero-E6 cells and A549 cells. BAPTA-AM-treated cells were incubated with HRB25 (M.O.I. = 10) and then assessed for viral RNA levels as described above. We found that the viral RNA level in BAPTA-AM-treated cells and control-treated cells were comparable, indicating that the cellular Ca^2+^ level has no effect on SARS-CoV-2 binding (S1A and S1B Fig). However, BAPTA-AM can significantly inhibit SARS-CoV-2 infection in Vero-E6 cells, indicating that the cellular Ca^2+^ level may affect SARS-CoV-2 infection after binding (S1C Fig).

**Fig 4:**
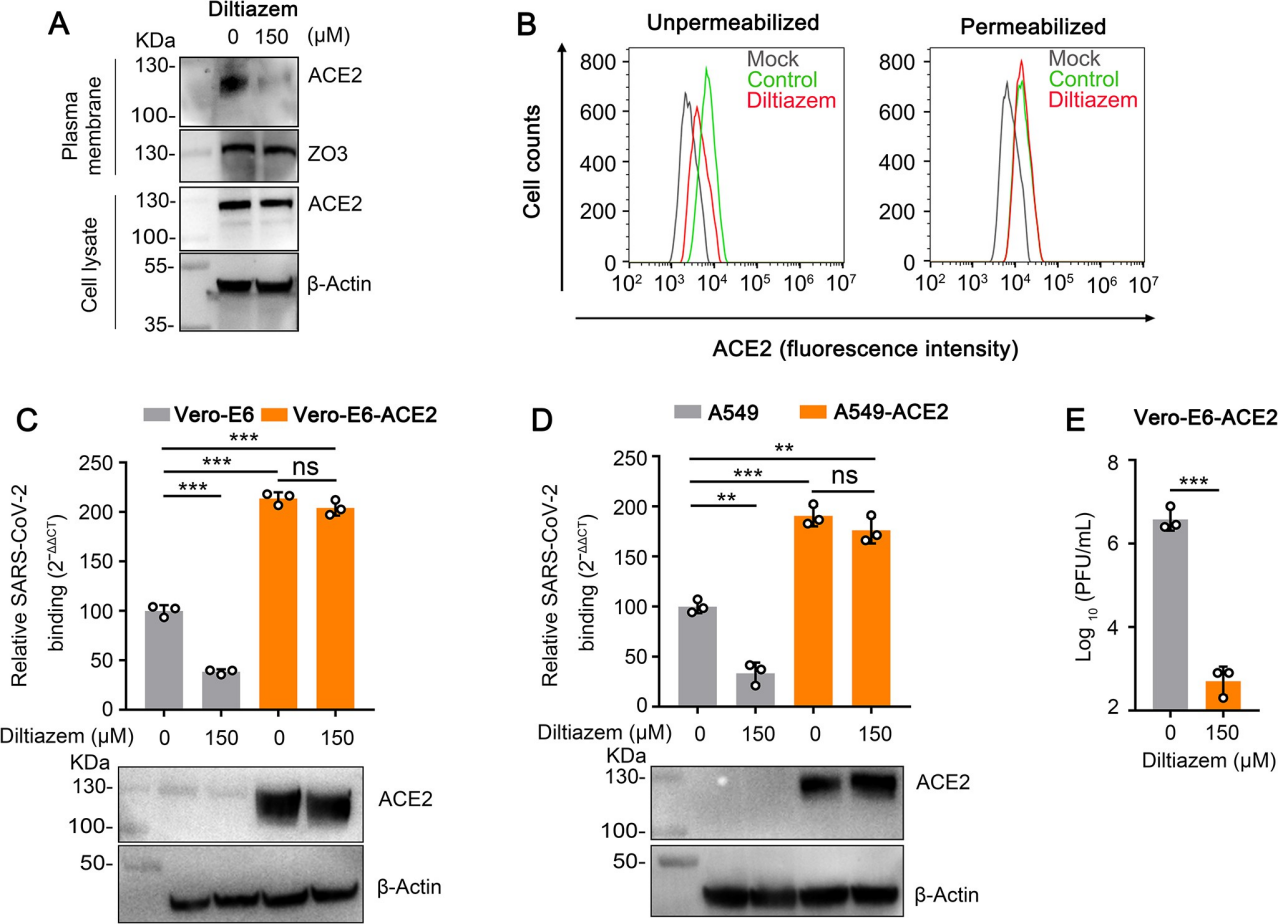
Diltiazem decreases the cell surface expression of ACE2. (A and B) Vero-E6 cells were preincubated with diltiazem, then the expression of ACE2 on the cell surface and in the total cells was detected by western blotting (A) and flow cytometry (B), respectively. (C and D) Diltiazem-treated Vero-E6 cells, Vero-E6-ACE2 cells (C), A549 cells, or A549-ACE2 cells (D) were incubated with HRB25 (M.O.I. = 10) at 4°C for 1 h, then washed with PBS. The viral RNA level in the cell lysate was measured by qPCR. The expression of exogenous ACE2 was confirmed by western blotting. (E) Diltiazem-treated Vero-E6-ACE2 cells were infected with HRB25 (M.O.I. = 0.01), and the supernatants were harvested at 24 h post-infection for plaque assays. The data shown represent, or are from, three independent experiments or replicates (means ± SDs). The two-tailed unpaired Student’s t-test was used for the statistical analysis. ns, not significant, **p < 0.01, ***p < 0.001.

Human ACE2 is a widely accepted binding receptor of SARS-CoV-2 [1,4,5,36]. We next tested whether diltiazem treatment inhibits the cell surface expression level of ACE2. Cell membrane proteins from diltiazem-treated Vero-E6 cells were western blotted to detect the cell surface expression of ACE2. Compared with mock-treated cells, diltiazem treatment decreased the cell surface expression of ACE2, whereas the total expression of ACE2 was comparable (Fig 4A). Flow cytometry confirmed the decrease in the cell surface expression of ACE2 in diltiazem-treated cells (Fig 4B). Of note, BAPTA-AM treatment had no effect on the cell surface expression of ACE2 in Vero-E6 cells (S2 Fig).

We next performed viral binding assays to test whether overexpression of ACE2 could overcome the inhibitory effect of diltiazem on SARS-CoV-2 binding. Vero-E6-ACE2 cells and A549-ACE2 cells, two cells generated with Vero-E6 cells and A549 cells to overexpress human ACE2, respectively, were treated with diltiazem and then infected with SARS-CoV-2 as described above. The viral RNA level in Vero-E6-ACE2 cells or A549-ACE2 cells was significantly higher than that in wild-type cells (Fig 4C and 4D). The viral RNA level of diltiazem-treated Vero-E6-ACE2 cells or A549-ACE2 cells was comparable to that of the mock-treated Vero-E6-ACE2 cells or A549-ACE2 cells, respectively (Fig 4C and 4D), indicating that overexpression of ACE2 can overcome the inhibition effect of diltiazem on SARS-CoV-2 binding. These results suggest that diltiazem treatment reduces the cell surface expression of ACE2 to inhibit SARS-CoV-2 infection.

We also performed a viral infection assay in Vero-E6-ACE2 cells to test whether overexpression of ACE2 could overcome SARS-CoV-2 infection. We found that diltiazem treatment decreased SARS-CoV-2 infection (Fig 4E), indicating that the overexpression of ACE2 could overcome the inhibitory effect on cell binding but not the viral infection after binding.

### Diltiazem inhibits the internalization of SARS-CoV-2

**Fig 5:**
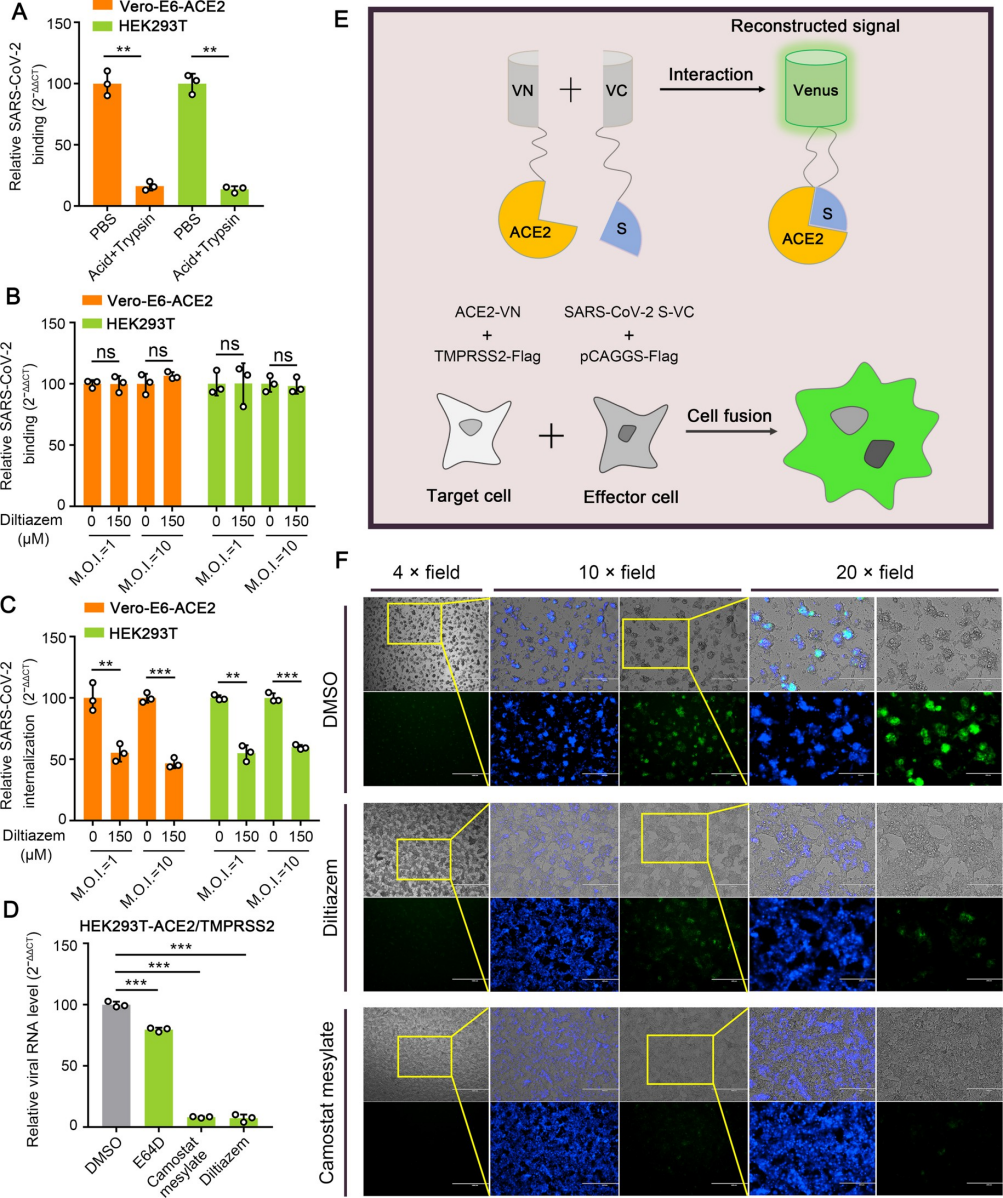
Diltiazem inhibits the internalization of SARS-CoV-2. (A) Cells were incubated with HRB25 (M.O.I. = 10) for 1 h at 4°C, then washed with acid buffer/trypsin to remove bound virus and lysed for qPCR to detect SARS-CoV-2. (B and C) Diltiazem-treated cells were incubated with HRB25 (M.O.I. = 1 or 10) at 4°C for 1 h and washed with PBS, and then shifted to 37°C for 1 h; they were then washed with PBS (B) or acid buffer/trypsin (C). The washed cells were lysed for qPCR to detect SARS-CoV-2 binding to cells (B) or internalized into cells (C). (D) HEK293T cells transiently expressing ACE2 and TMPRSS2 were treated with DMSO, E64D, camostat mesylate, or diltiazem, and then infected with HRB25 (M.O.I. = 5). The viral RNA level in the cell lysate was measured by qPCR at 6 h post-infection. (E) Schematic representation of the SARS-CoV-2 S protein-mediated cell-cell fusion assay. (F) Representative images of DMSO-, camostat mesylate-, or diltiazem-treated cell-cell fusion. Scale bar: 4 × field, 1000 μm; 10 × field, 400 μm; and 20 × field, 200 μm. The data shown are the means ± SDs of three independent experiments or replicates. The two-tailed unpaired Student’s t-test was used for the statistical analysis. ns, not significant, **p < 0.01, ***p < 0.001.

SARS-CoV-2 is internalized into cells through the receptor-mediated endocytosis pathway following fusion at the endosome that is mediated by endosomal cysteine protease cathepsin L or through direct fusion at the plasma membrane in the presence of TMPRSS2 [4,37]. SARS-CoV-2 mainly enters Vero-E6 cells or HEK293T cells through receptor-mediated endocytosis following fusion at the endosome [4,38,39]. We therefore performed a viral internalization assay to test whether diltiazem inhibits the endocytosis of SARS-CoV-2 at an M.O.I. of 10 or 1 following viral attachment in Vero-E6-ACE2 cells and HEK293T cells. After being incubated with SARS-CoV-2 at 4°C for 1 h, the washed cells were shifted to 37°C for 1 h to allow the internalization of bound viruses. The cells were washed with normal PBS or acid buffer/trypsin, which efficiently removes cell surface-bound SARS-CoV-2 (Fig 5A). The washed cells were lysed for qPCR to detect SARS-CoV-2. We found that diltiazem treatment strongly inhibited SARS-CoV-2 internalization into Vero-E6-ACE2 cells and HEK293T cells at high and low infection doses while having no effect on SARS-CoV-2 binding to these two cell types (Fig 5B and 5C).

In HEK293T-ACE2/TMPRSS2 cells, SARS-CoV-2 enters mainly through TMPRSS2-dependent membrane fusion [4]. We therefore investigated whether diltiazem inhibits TMPRSS2-mediated fusion during SARS-CoV-2 infection in HEK293T-ACE2/TMPRSS2 cells. Cells were treated with DMSO, E64D, camostat mesylate, which inhibits TMPRSS2 [40], or diltiazem for 1 h, and were then infected with HRB25 (M.O.I. = 5). The viral RNA level in the cell lysate relative to that of 28S rRNA was determined by qPCR at 6 h post-infection. The results showed that the viral RNA level in diltiazem-treated cells was significantly lower than that in DMSO-treated cells at 6 h post-infection, indicating that diltiazem affects TMPRSS2-dependent membrane fusion during SARS-CoV-2 infection (Fig 5D).

We confirmed the above result by using a microscopy-based assay. We generated a SARS-CoV-2 S protein-mediated cell-cell fusion system by using a bimolecular fluorescence complementation system, in which two cells separately express half of the green fluorescent protein Venus will produce Venus only upon fusion. HEK293T cells co-transfected with ACE2-VN and TMPRSS2-Flag plasmids were used as the target cells, and HEK293T cells co-transfected with SARS-CoV-2 S-VC and pCAGGS-Flag were used as the effector cells (Fig 5E). After the effector cells and target cells were cocultured for 18 h at 37°C, syncytia containing multiple nuclei and green fluorescence could be easily observed under light and fluorescence microscopy, respectively (Fig 5F). Compared with DMSO-treated cells, diltiazem or camostat mesylate treatment significantly reduced the intensity of green fluorescence and inhibited syncytium formation (Fig 5F), confirming that diltiazem inhibits TMPRSS2-dependent membrane fusion during SARS-CoV-2 infection. These results demonstrate that diltiazem inhibits SARS-CoV-2 infection by affecting internalization.

### Knockdown of Ca_v_1.2 α_1c_ inhibits the attachment and internalization of SARS-CoV-2

**Fig 6:**
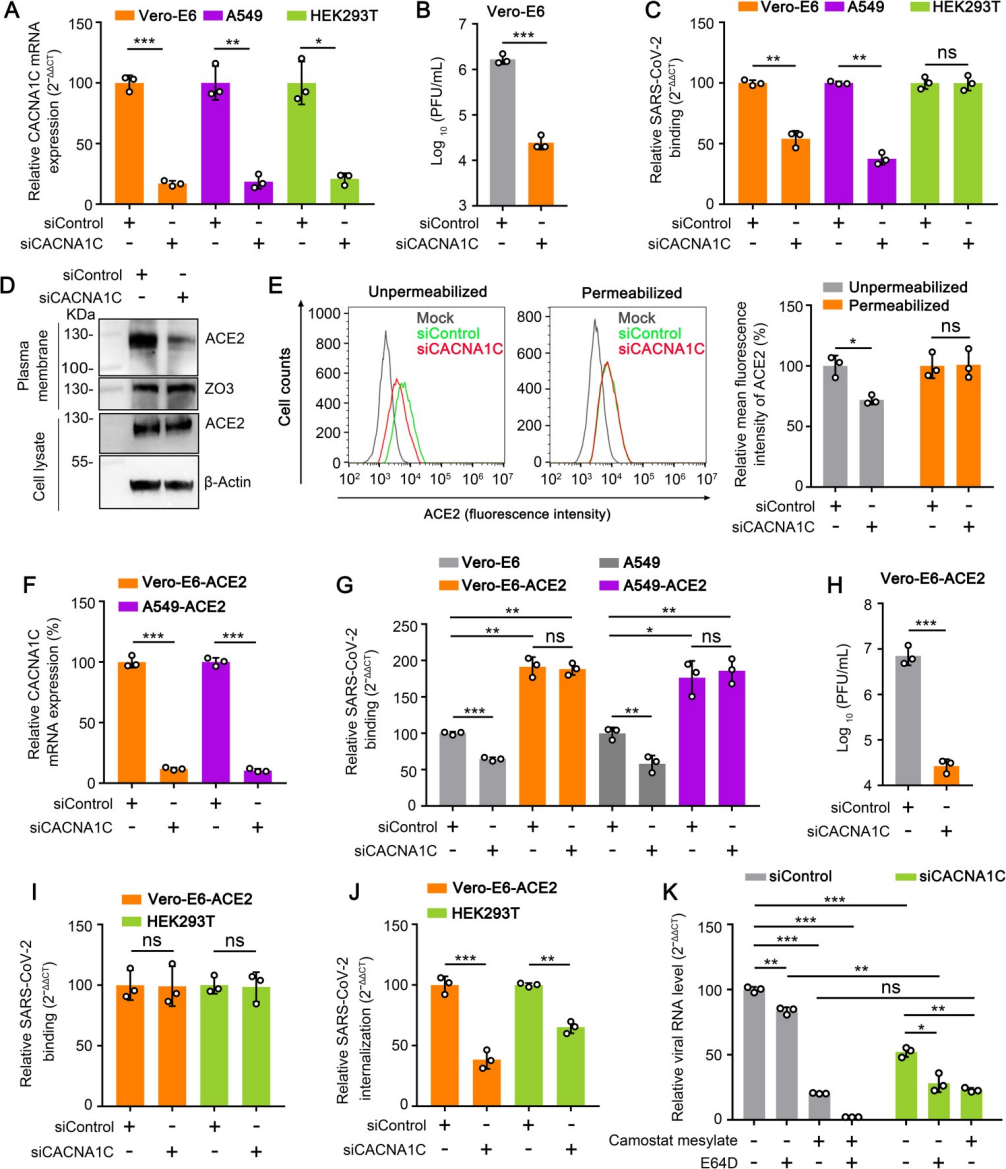
Knockdown of Ca_v_1.2 α_1c_ inhibits the attachment and internalization of SARS-CoV-2. (A) The CACNA1C mRNA level in the indicated siRNA-transfected cells was measured by qPCR. siControl, scrambled siRNA. siCACNA1C, siRNA specific for CACNA1C mRNA. (B) CACNA1C-silenced Vero-E6 cells were infected with HRB25 (M.O.I. = 0.01), and the supernatants were harvested at 24 h post-infection for plaque assays. (C) CACNA1C-silenced cells were incubated with HRB25 (M.O.I. = 10) for 1 h at 4°C. The viral RNA level in the cell lysate was measured by qPCR. (D and E) The expression of ACE2 on the cell surface and in total cells was detected by western blotting (D) and flow cytometry (E) in CACNA1C-silenced Vero-E6 cells. (F) The CACNA1C mRNA level in the indicated siRNA-transfected cells was measured by qPCR. (G) CACNA1C-silenced cells were incubated with HRB25 (M.O.I. = 10) for 1 h at 4°C, and washed with PBS. The viral RNA level in the cell lysate was measured by qPCR. (H) CACNA1C-silenced Vero-E6-ACE2 cells were infected with HRB25 (M.O.I. = 0.01), and the supernatants were harvested at 24 h post-infection for plaque assays. (I and J) CACNA1C-silenced cells were incubated with HRB25 (M.O.I. = 10) at 4°C for 1 h and washed with PBS, then shifted to 37°C for 1 h. The cells were then washed with PBS (I) or acid buffer/trypsin (J). The washed cells were lysed for qPCR to detect SARS-CoV-2 binding to cells (I) or internalized into cells (J). (K) DMSO-, E64D-, camostat mesylate-, or diltiazem-treated CACNA1C-silenced HEK293T cells transiently expressing ACE2 and TMPRSS2 were infected with HRB25 (M.O.I. = 5). The viral RNA level in the cell lysate was measured by qPCR at 6 h post-infection. The data shown are the means ± SDs of three independent experiments or replicates. The two-tailed unpaired Student’s t-test was used for the statistical analysis. ns, not significant, *p < 0.05, **p < 0.01, ***p < 0.001.

Diltiazem is a functional antagonist of calcium channels and mainly targets Ca_v_1.2 α_1c_, a typical cytomembrane-bound protein coded by the calcium voltage-gated channel subunit alpha 1 C gene (CACNA1C) [41,42]. To investigate whether diltiazem inhibits SARS-CoV-2 infection by targeting Ca_v_1.2 α_1c_, we first performed an RNAi assay in Vero-E6 cells to detect whether Ca_v_1.2 α_1c_ is required for SARS-CoV-2 infection. Cells were transfected with siRNA targeting CACNA1C, and were then infected with HRB25 (M.O.I. = 0.01) at 72 h post-transfection. Infectious titers in the supernatants of the infected cells were detected by plaque assay at 24 h post-infection. qPCR analysis confirmed that CACNA1C mRNA expression was significantly reduced in Vero-E6 cells at 12 h post-transfection (Fig 6A). Compared to siControl-transfected cells, knockdown of CACNA1C significantly decreased the viral titers in the supernatant (Fig 6B), indicating that Ca_v_1.2 α_1c_ is required for SARS-CoV-2 infection.

We then performed an RNAi assay to determine whether CACNA1C affects SARS-CoV-2 binding. qPCR analysis also confirmed that CACNA1C mRNA expression was significantly reduced in A549 cells and HEK293T cells at 24 h post-transfection (Fig 6A). CACNA1C-silenced Vero-E6 cells, A549 cells, and HEK293T cells were treated as described above and the viral RNA level in the cell lysate relative to the RNA level of β-actin (Vero-E6 cells) or 28S rRNA (A549 cells and HEK293T cells) was detected by qPCR. Compared to the viral RNA level of siControl-transfected cells, the viral RNA level in CACNA1C-silenced Vero-E6 cells and A549 cells was significantly decreased (Fig 6C), whereas the viral RNA level in CACNA1C-silenced HEK293T cells was comparable to that of siControl-transfected cells (Fig 6C), indicating that Ca_v_1.2 α_1c_ is important for SARS-CoV-2 binding to cells in a cell type-dependent manner.

We next tested whether knockdown of CACNA1C affects the cell surface expression of ACE2. Cell membrane proteins from CACNA1C-silenced Vero-E6 cells were western blotted to detect the expression of ACE2. The cell surface expression of ACE2 in CACNA1C-silenced cells was significantly lower than that of siControl-transfected Vero-E6 cells; however, the total expression of ACE2 in CACNA1C-silenced cells was comparable to that of siControl-transfected Vero-E6 cells (Fig 6D). Flow cytometry further confirmed this observation (Fig 6E). These results indicate that knockdown of Ca_v_1.2 α_1c_ affects the cell surface expression of ACE2.

To test whether overexpression of ACE2 can overcome the inhibitory effect of Ca_v_1.2 α_1c_ knockdown on SARS-CoV-2 binding, we performed viral binding assays in CACNA1C-silenced Vero-E6-ACE2 cells and A549-ACE2 cells as described above. We first confirmed that CACNA1C mRNA transcription is significantly decreased in Vero-E6-ACE2 cells and A549-ACE2 cells at 12 h and 24 h after transfection, respectively (Fig 6F). Then, CACNA1C-silenced cells were incubated with SARS-CoV-2 for the binding assay. The viral RNA level of CACNA1C-silenced Vero-E6-ACE2 cells and A549-ACE2 cells was comparable to that of the siControl-transfected Vero-E6-ACE2 cells and A549-ACE2 cells but significantly higher than that of CACNA1C-silenced Vero-E6 cells and A549 cells, respectively (Fig 6G). These results indicate that overexpression of ACE2 can overcome the inhibitory effect of knockdown of Ca_v_1.2 α_1c_ on the cell binding of SARS-CoV-2.

Of note, the viral titers in the supernatant of CACNA1C-silenced Vero-E6-ACE2 cells were still significantly lower than those of siControl-transfected Vero-E6-ACE2 cells (Fig 6H). This result indicates that overexpression of ACE2 cannot overcome the inhibitory effect of Ca_v_1.2 α_1c_ knockdown on SARS-CoV-2 infection. Ca_v_1.2 α_1c_ may affect the stages of SARS-CoV-2 infection after binding. We tested whether Ca_v_1.2 α_1c_ is required for internalization of SARS-CoV-2 in Vero-E6-ACE2 cells as described above. We found that knockdown of Ca_v_1.2 α_1c_ strongly inhibited SARS-CoV-2 internalization into Vero-E6-ACE2 cells, while having no effect on SARS-CoV-2 binding (Fig 6I and 6J). Similar to the effects seen with diltiazem treatment, knockdown of Ca_v_1.2 α_1c_ strongly inhibited SARS-CoV-2 internalization into HEK293T cells, while having no effect on SARS-CoV-2 binding (Fig 6I and 6J). We also confirmed that knockdown of Ca_v_1.2 α_1c_ affects TMPRSS2-dependent membrane fusion during SARS-CoV-2 infection in HEK293T-ACE2/TMPRSS2 cells by qPCR detection. In HEK293T-ACE2/TMPRSS2 cells, the viral RNA level in CACNA1C-silenced cells was lower than that in siControl-transfected cells at 6 h post-infection (Fig 6K). These results indicate that diltiazem inhibits the binding and internalization of SARS-CoV-2 by targeting Ca_v_1.2 α_1c_.

### Ca_v_1.2 α_1c_ interacts and colocalizes with SARS-CoV-2 S protein and ACE2

**Fig 7:**
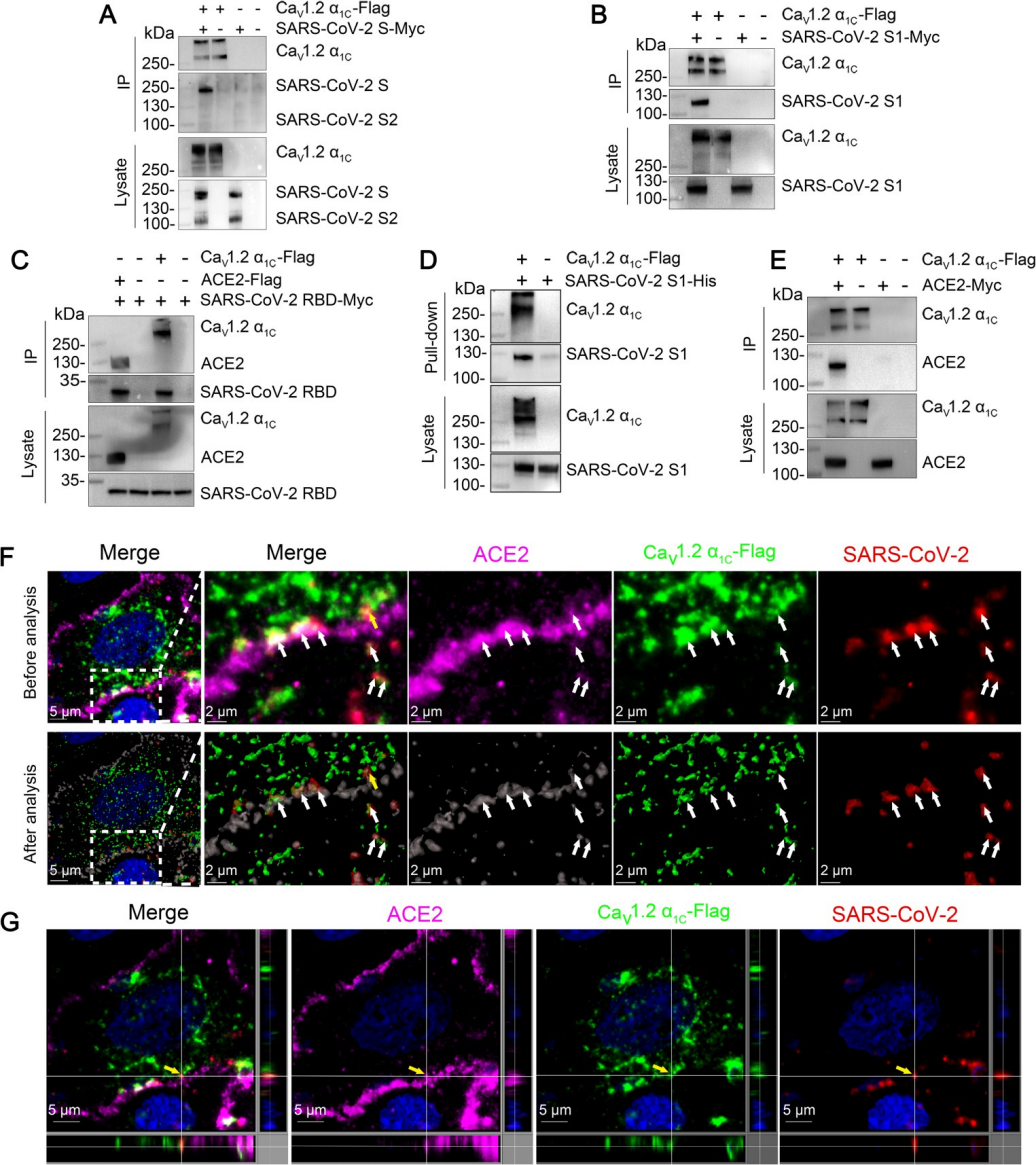
Ca_v_1.2 α_1c_ interacts and colocalizes with SARS-CoV-2 S protein and ACE2. (A) HEK293 cells were co-transfected with Ca_v_1.2 α_1c_-Flag and SARS-CoV-2 S-Myc, and then subjected to immunoprecipitation (IP) by using anti-Flag agarose beads. Representative western blots of whole-cell lysates and eluates after IP are shown. (B) The co-immunoprecipitation of Ca_v_1.2 α_1c_-Flag and the SARS-CoV-2 S protein truncation mutant SARS-CoV-2 S1-Myc. (C) The co-immunoprecipitation of Ca_v_1.2 α_1c_-Flag and ACE2-Flag with the SARS-CoV-2 S protein truncation mutant SARS-CoV-2 RBD-Myc. (D) Purified soluble His-tagged SARS-CoV-2 S1 protein was pooled with the lysate from Ca_v_1.2 α_1c_-Flag transfected HEK293 cells and then pulled-down by using anti-Flag agarose beads. (E) The co-immunoprecipitation of Ca_v_1.2 α_1c_-Flag and ACE2-Myc. (F and G) Immunofluorescence assay. Vero-E6 cells were transfected with Ca_v_1.2 α_1c_-Flag for 24 h, then cells were incubated with SARS-CoV-2 (M.O.I. = 10) at 4°C for 1 h. The images comprising three single fluorescence channels were analysis by using Imaris software. The arrowhead indicates the representative colocalization of Ca_v_1.2 α_1c_-Flag (green), ACE2 (purple), and SARS-CoV-2 nucleocapsid protein (red) (F). The colocalization of Ca_v_1.2 α_1c_-Flag, ACE2, and SARS-CoV-2 nucleocapsid protein (indicated by the yellow arrowhead) is shown in three dimensions (G). The data shown are representative of three independent experiments.

SARS-CoV-2 S protein is responsible for SARS-CoV-2 binding and internalization. Therefore, we performed a co-immunoprecipitation assay to examine whether Ca_v_1.2 α_1c_ interacts directly with SARS-CoV-2 S protein. Flag-tagged Ca_v_1.2 α_1c_ protein (Ca_v_1.2 α_1c_-Flag) was co-expressed with Myc-tagged SARS-CoV-2 S protein (SARS-CoV-2 S-Myc) in plasmid-transfected HEK293 cells. Immunoblotting for SARS-CoV-2 S-Myc demonstrated that Ca_v_1.2 α_1c_ interacts with SARS-CoV-2 S protein specifically, but barely interacts with the SARS-CoV-2 S2 subdomain (Fig 7A), indicating that Ca_v_1.2 α_1c_ interacts with the SARS-CoV-2 S1 subdomain. We then performed a co-immunoprecipitation analysis by using the S1 subdomain or RBD of SARS-CoV-2 S protein in plasmid-transfected HEK293 cells and found that both the S1 subdomain and RBD interact with Ca_v_1.2 α_1c_ (Fig 7B and 7C).

Next, we performed a pull-down assay by using purified SARS-CoV-2 S1 subdomain (SARS-CoV-2 S1-His) and cell lysate from Ca_v_1.2 α_1c_-Flag-transfected HEK293 cells. Ca_v_1.2 α_1c_ successfully pulled down the SARS-CoV-2 S1 subdomain (Fig 7D), indicating that Ca_v_1.2 α_1c_ directly interacts with the SARS-CoV-2 S1 subdomain. Of note, we also found that Ca_v_1.2 α_1c_ interacts with ACE2 in a co-immunoprecipitation assay (Fig 7E). We further incubated virus with cells at 4°C and then performed an immunofluorescence assay to investigate whether Ca_v_1.2 α_1c_ colocalizes with SARS-CoV-2 and ACE2 in Ca_v_1.2 α_1c_-Flag-transfected Vero-E6 cells. We found that Ca_v_1.2 α_1c_-Flag does indeed colocalize with SARS-CoV-2 and ACE2 (Fig 7F and 7G).

### Diltiazem inhibits the replication of SARS-CoV-2 in mouse lung

**Fig 8:**
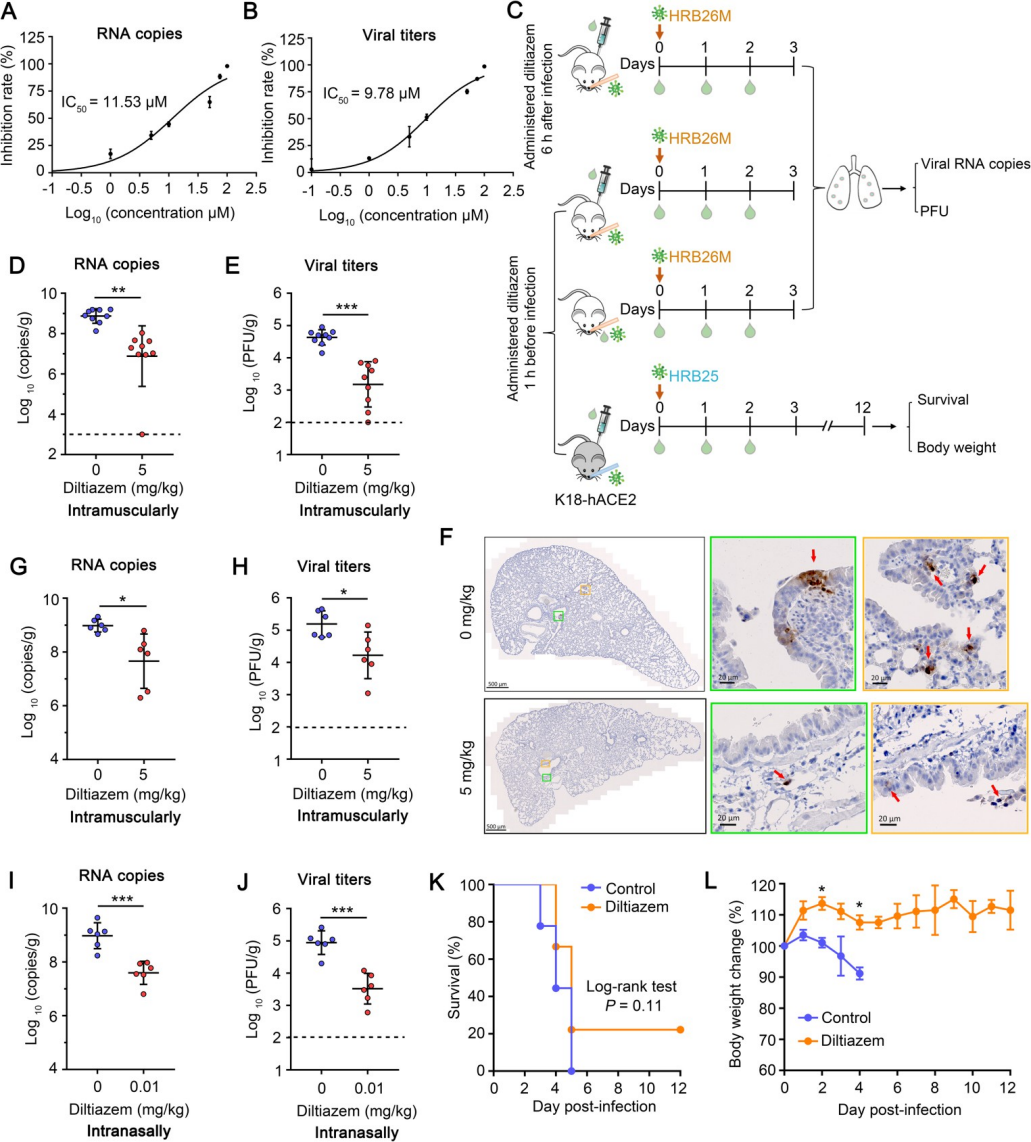
Diltiazem inhibits the replication of SARS-CoV-2 in mouse lung. (A and B) IC_50_ of diltiazem to HRB26M infectivity. Vero-E6 cells were treated with diltiazem at different concentrations or with vehicle (water) for 1 h at 37°C, and then infected with HRB26M (M.O.I. = 0.01), the supernatants were harvested at 24 h post-infection to determine viral RNA copy numbers (A) and viral titers (B) by using qPCR and plaque assays, respectively. (C) Protocol for *in vivo* experiments with BALB/c mice (white) and K18-hACE2 mice (black). (D and E) The mice were intramuscularly administered diltiazem (5 mg/kg) starting 1 h before being infected with HRB26M. The infectious viruses in the lungs of mice (n = 9) were measured by qPCR (D) and plaque assay (E) respectively. (F) Viral antigen in mouse lungs was detected by using an IHC assay. The red arrowhead indicates the representative viral antigen signals. (G and H) The mice were intramuscularly administered diltiazem (5 mg/kg) starting 6 h after being infected with HRB26M. The infectious viruses in the lungs of mice (n = 6) were detected as described in (D) and (E). (I and J) The mice were intranasally administered diltiazem (0.01 mg/kg) starting 1 h before being infected with HRB26M. The infectious viruses in the lungs of mice (n = 6) were detected as described in (D) and (E). (K and L) K18-hACE2 mice were intramuscularly administrated diltiazem (5 mg/kg) starting 1 h before being infected with HRB25. The survival (K) and body weight (L) of the mice (n = 9) were monitoring for 12 days after infection. The horizontal dashed lines indicate the limit of detection. The data shown in panels D and E represent the sum of three independent experiments, those in panels G–L represent the sum of two independent experiments. The two-tailed unpaired Student’s t-test was used for the statistical analysis, mean ± SDs, *p < 0.05, ***p < 0.001.

Finally, we tested whether diltiazem could inhibit the replication of SARS-CoV-2 *in vivo* by using BALB/c mice challenged with a mouse-adapted SARS-CoV-2 (HRB26M) [43]. HRB26M can efficiently replicate in the upper and lower respiratory tracts of BALB/c mice and C57BL/6J mice and is generally cleared after a couple of days. We first confirmed that HRB26M shows similar sensitivity to diltiazem as HRB25 in Vero-E6 cells (Fig 8A and 8B). After that, 6-week-old BALB/c mice were intramuscularly inoculated with diltiazem at a dose of 5 mg/kg. One hour later, mice were intranasally infected with HRB26M at a dose of 30 plaque form unit (PFU). Viral RNA and infectious viruses in the lungs were assessed at 3 days post-infection by use of qPCR and plaque assays, respectively (Fig 8C). Compared to mock-treated mice, diltiazem-treated mice had, on average, 100-times lower viral RNA copy numbers and 30-times lower viral titers in their lung tissues (Fig 8D and 8E). Immunohistochemistry (IHC) confirmed the results from the qPCR and plaque assays in the lungs (Fig 8F). The results suggest that diltiazem treatment significantly decreases viral replication in the lungs of mice.

We then tested whether diltiazem has a therapeutic effect on SARS-CoV-2 infection. HRB26M-infected mice were intramuscularly inoculated with diltiazem at a dose of 5 mg/kg at 6 h post-infection. Viral RNA and infectious viruses in the lungs were assessed at 3 days post-infection by using qPCR and plaque assays, respectively. Compared to mock-treated mice, diltiazem-treated mice had on average 21-times lower viral RNA copy numbers and 9-times lower viral titers in their lung tissues (Fig 8G and 8H), indicating that diltiazem has potential therapeutic efficacy against SARS-CoV-2 infection.

We next tested the viral inhibition effect of diltiazem upon intranasal delivery. Six-week-old BALB/c mice were intranasally inoculated with diltiazem at a dose of 0.01 mg/kg, and were infected with HRB26M 1 h later. The viral RNA and infectious viruses in the lungs were assessed at 3 days post-infection by using qPCR and plaque assays, respectively. Compared to mock-treated mice, diltiazem-treated mice had, on average, 24-times lower viral RNA copy numbers and 27-times lower viral titers in their lung tissues (Fig 8I and 8J). The effect of intranasal inoculation was thus similar to that of intramuscular injection.

We further examined the protective effects of diltiazem in the human ACE2 transgenic C57BL/6J mouse (K18-hACE2 mouse). Infection of K18-hACE2 mice with SARS-CoV-2 resulted in a dose-dependent increase in weight loss and mortality with lung lesions and immune cell infiltration. However, the enhanced penetrance of central nervous system and virus replication in central nervous system are considered as important contribution to mortality [44,45]. Six-week-old K18-hACE2 mice were intramuscularly inoculated with diltiazem at a dose of 5 mg/kg. One hour later, these mice were intranasally infected with HRB25 at a dose of 100 PFU (Fig 8C). Corresponding body weight changes and survival were observed for 12 days. By 3-days post-infection, 2 of the 9 mock-treated mice had died and the remaining mice were lethargic and starting to loss body weight (Fig 8K and 8L). By 5-days post-infection, all of the mock-treated mice had succumbed to their infection (Fig 8K). Diltiazem-treated mice had no significant body weight loss during the observation period and appeared healthy at 3-days post-infection (Fig 8L). By 5-days post-infection, seven diltiazem-treated mice had succumbed to their infection but two diltiazem-treated mice survived to the end of the observation period (12-days post-infection) (Fig 8K). These results indicate that diltiazem inhibits viral replication in the lungs of BALB/c mice. In addition, the diltiazem protects more than 20% of K18-hACE2 mice from death due to SARA-CoV-2 infection, although there was no statistically significant difference between diltiazem-treated and control K18-hACE2 mice.

## Discussion

In this study, we found that diltiazem, a blocker of Ca_v_1.2 α_1c_ and an FDA-approved drug, inhibits cell binding and internalization of SARS-CoV-2, and decreases SARS-CoV-2 infection in cells and mice. Knockdown of Ca_v_1.2 α_1c_ showed similar effects to diltiazem treatment in terms of cell attachment and internalization of SARS-CoV-2. Ca_v_1.2 α_1c_ was found to interact with SARS-CoV-2 S protein and ACE2. Our findings suggest that diltiazem has potential as a drug against SARS-CoV-2 infection and that Ca_v_1.2 α_1c_ is a promising target for antiviral drug development for COVID-19.

An ideal drug to treat COVID-19 should be safe, affordable, and accessible. Unprecedented worldwide efforts have been made to identify new drugs and repurpose approved drugs as anti-SARS-CoV-2 therapeutics, including remdesivir, lopinavir, ritonavir, ribavirin, arbidol, ostalmovir, and favipiravir, which have been found to be effective [46–48]. Remdesivir is the only authorized drug approved by the US FDA for emergency use, even though it has little effect on hospitalized patients with COVID-19 [49]. The administration of ribavirin has been reported to cause elevated alanine transferase levels and hepatic toxicity in huge numbers of patients [47,48]. Similar side effects have been reported upon administration of lopinavir, ritonavir, favipiravir, and arbidol [47,50]. Diltiazem belongs to the benzothiazepine class of calcium channel blockers and is an effective blocker of different L-type calcium channels. Its IC_50_ is approximately 45 μM for the Ca_v_1.2 L-type calcium channel, 326 μM for the Ca_v_1.3 L-type calcium channel, and 92 μM for the Ca_v_1.4 L-type calcium channel [41,51,52]. The IC_50_ of diltiazem for SARS-CoV-2 infection and entry is about 10 μM (S3 Fig), far below the IC_50_ values for Ca_v_1.2, Ca_v_1.3, and Ca_v_1.4 L-type calcium channels, which suggests that diltiazem could be a safe antiviral drug candidate for SARS-CoV-2 infection. Diltiazem was approved in the US in 1982 [53]. It is cheap and widely used in clinical practice for many indications, including atrial arrhythmia, hypertension, and angina [28,54,55]. Clinically, diltiazem is administered orally or intravenously. The oral dose for adults is about 120–480 mg/day and the dose for continuous intravenous infusion is about 120–240 mg/day [56]. Although, the toxic dose in human is not known, the oral 50% lethal dose (LD_50_) is 415–740 mg/kg in mice, 560–810 mg/kg in rats, and >50 mg/kg in dogs; the intravenous LD_50_ is 60 mg/kg in mice and 38 mg/kg in rats [57]. In our study, we found that whether 1 h before or 6 h after SARS-CoV-2 exposure, an intramuscular dose of 5 mg/kg can significantly decrease SARS-CoV-2 infection in mice, which is far below the toxicity doses just listed. Furthermore, when given 1 h before SARS-CoV-2 exposure, an intranasal diltiazem dose of 0.01 mg/kg also can significantly decrease SARS-CoV-2 infection in mice. These results further champion diltiazem as a potential prophylactic or therapeutic against SARS-CoV-2 infection.

Our results do not exclude the possibility that diltiazem may affect other stages of SARS-CoV-2 infection. Previous studies have shown that disrupting cellular calcium levels reduces the fusion of SARS-CoV and MERS-CoV with host cells [21,22], and disruption of Ca_v_1.2 α_1c_ expression also alters cellular calcium levels [58]. Whether Ca_v_1.2 α_1c_ affects other stages of SARS-CoV-2 infection after cell binding and internalization remains to studied. Given that SARS-CoV-2 is prone to mutate over time [59], it would be beneficial if diltiazem affects other stages of SARS-CoV-2 infection, because that might limit the emergence of diltiazem-resistant SARS-CoV-2 variants.

Ca_v_1.2 α_1c_ represents a novel cellular membrane protein that is important for SARS-CoV-2 infection. Our results demonstrate that knockdown of Ca_v_1.2 α_1c_ leads to similar effects as those induced by diltiazem treatment in cells, that is, reducing SARS-CoV-2 cell binding and internalization. Moreover, our study shows that Ca_v_1.2 α_1c_ interacts with the RBD of the S protein and ACE2. Taken together, our results indicate that diltiazem inhibits SARS-CoV-2 infection at least in part by blocking its effector molecule Ca_v_1.2 α_1c_ and thereby affecting the cell binding and internalization of the virus. A recent preprint reported that felodipine, another L-type calcium channel blocker, has an inhibitory effect on SARS-CoV-2 infection of cells [60], which suggests that multiple members of the L-type calcium channel family play an important role in SARS-CoV-2 infection, and could be targets for the development of anti-SARS-CoV-2 reagents.

Understanding how Ca_v_1.2 α_1c_ affects SARS-CoV-2 infection is important to develop drugs against COVID-19 by targeting Ca_v_1.2 α_1c_. There are several possible mechanisms for Ca_v_1.2 α_1c_-promoted SARS-CoV-2 binding. One possibility is that Ca_v_1.2 α_1c_ affects SARS-CoV-2 binding by regulating the cell surface expression of ACE2. We found that diltiazem treatment or knockdown of Ca_v_1.2 α_1c_ decreased the cell surface expression of ACE2 in cell lines that highly express ACE2 naturally. Overexpression of ACE2 can rescue the inhibitory effect of diltiazem treatment or Ca_v_1.2 α_1c_ knockdown on SARS-CoV-2 binding. However, we also found that Ca_v_1.2 α_1c_ affects SARS-CoV-2 binding in cells that express ACE2 at a low level. Whether Ca_v_1.2 α_1c_ affects SARS-CoV-2 binding by regulating cell surface ACE2 levels in the cells with low ACE2 expression needs further investigation. Another possibility is that Ca_v_1.2 α_1c_ may enhance SARS-CoV-2 binding directly by forming a complex with the S protein, ACE2, and potentially other molecules during infection. We found that Ca_v_1.2 α_1c_ interacts with the RBD of the S protein and ACE2. A recent study reported that heparan sulfate (HS) acts as a cell attachment factor for SARS-CoV-2 infection [14]. HS interacts with the RBD of SARS-CoV-2 S protein and ACE2, and enhances the interaction between the two by increasing the “up” conformation of the RBD. Interestingly, it had been reported that Ca_v_1.2 α_1c_ also directly interacts with HS through the first pore-forming domain of Ca_v_1.2 α_1c_ [61]_._ It may be that the trimetric S protein of SARS-CoV-2, together with HS, ACE2, and Ca_v_1.2 α_1c_, forms a much more stable complex that facilitates cell binding and infection. Notably, diltiazem treatment or knockdown of Ca_v_1.2 α_1c_ does not decrease SARS-CoV-2 binding but affects internalization in HEK293T cells. Therefore, inhibition of binding does not appear to be the predominate mechanism for Ca_v_1.2 α_1c_ to affect SARS-CoV-2 infection.

Diltiazem inhibits SARS-CoV-2 internalization, indicating that activated Ca_v_1.2 α_1c_ is required for SARS-CoV-2 internalization and Ca^2+^-signaling may be involved in this process. Ca^2+^ influx can transiently increase the concentration of Ca^2+^ around Ca^2+^-sensitive processes (local Ca^2+^-signaling) or robustly increase the concentration of Ca^2+^ throughout the entire cell [62]. BAPTA-AM treatment affects SARS-CoV-2 infection but not binding or internalization (S1D and S1E Fig), which indicates that the entire cell Ca^2+^ level affects SARS-CoV-2 infection after internalization. A previous study found that Ca_v_1.2 α_1c_ interacts with influenza virus hemagglutinin and mediates virus entry into host cells [24]. Influenza virus entry can induce a small, transient increase in the concentration of Ca^2+^ in areas where virus particles have been adsorbed prior to the robust increase in the entire cell Ca^2+^ level [24]. Whether local Ca_v_1.2-dependent Ca^2+^-signaling is similarly involved in the internalization process of SARS-CoV-2 remains to be investigated.

## Materials and methods

### Ethics statement

All 4-6-week-old female BALB/c mice were purchased from Beijing Vital River Laboratory Animal Technology. Human ACE2 transgenic C57BL/6J (K18-hACE2) mice were purchased from GemPharmaTech. Mice were maintained under conventional conditions in the animal biosafety level 4 facilities at the HVRI of CAAS, which is approved for such use by the Ministry of Agriculture and Rural Affairs of China. All institutional and national guidelines for the care and use of laboratory animals were followed. All mouse experiments were carried out in strict accordance with the recommendations in the Guide for the Care and Use of Laboratory Animals of the Ministry of Science and Technology of the People’s Republic of China. The protocols were approved by the Committee on the Ethics of Animal Experiments of the HVRI of CAAS (Approval number 2020-01-01JiPi).

### Cell lines

HEK293 cells (ATCC, CRL-1573), HEK293T cells (ATCC, CRL-3216), Vero-E6 cells (ATCC, CRL-1586), and HeLa cells (ATCC-CCL-2) were maintained in Dulbecco’s modified Eagle’s medium (DMEM), A549 cells (ATCC, CCL-185) were maintained in F-12K Nutrient Mixture, supplemented with 10% fetal bovine serum (FBS), 1% penicillin/streptomycin, and L-glutamine at 37°C in 5% CO_2_. Calu-3 cells (ATCC, HTB-55) were grown in Eagle’s minimal essential medium supplemented with 10% FBS, 1% penicillin/streptomycin, L-glutamine, and 0.1 mM non-essential amino acids at 37°C in 5% CO_2_. BEAS-2B cells were obtained from the Conservation Genetics CAS Kunming Cell Bank, and maintained in Bronchial Epithelial Cell Growth Basal Medium (Lonza, Switzerland). Vero-E6 cells and A549 cells expressing human ACE2 (Vero-E6-ACE2 cells, A549-ACE2 cells) were generated by transducing an ACE2-expressing lentiviral vector, and selecting with puromycin, after selecting, cells were subsequently maintained with puromycin.

### Viruses

SARS-CoV-2/HRB25/human/2020/CHN (HRB25, GISAID access no. EPI_ISL_467430), mouse-adapted SARS-CoV-2/HRB26/human/2020/CHN (HRB26M, GISAID access no. EPI_ISL_459910) were maintained in our laboratory [32,43]. All experiments with infectious SARS-CoV-2 were performed in the biosafety level 4 and animal biosafety level 4 facilities at the Harbin Veterinary Research Institute (HVRI) of the Chinese Academy of Agricultural Sciences (CAAS).

### Plasmids

The SARS-CoV-2 S (GenBank: MN908947.3), S1 subunit (aa 14–685) and RBD (aa 331–524) were cloned into the pCAGGS-Myc vector. Human Ca_v_1.2 α_1c_, ACE2, and TMPRSS2 cDNAs were cloned into the pCAGGS-Flag vector and confirmed by sequencing analysis.

To construct the bimolecular fluorescence complementation system expression vectors, the C-terminal of ACE2 was fused in-frame with the N-terminal 2–173 amino acids of the green fluorescent protein Venus (ACE2-VN), and the C-terminal of SARS-CoV-2 S was fused with the C-terminal 154–238 amino acids of Venus (SARS-CoV-2 S-VC).

### Plaque assay

Serial dilutions of supernatants from infected cells or animal tissues were added to Vero-E6 cell monolayers and adsorbed for 1 h at 37°C. The cells were then washed and plaque media was overlaid on them before they were incubated at 37°C. After 48 h of incubation, the cell monolayers were stained with crystal violet and the plaques were counted.

### Real-time quantitative PCR (qPCR)

The viral RNA copies in the samples collected from the cells and animals were determined as described previously [63]. Total RNA was isolated with TRIzol reagent (Invitrogen), 1 μg of total RNA was used for reverse transcription (Vazyme). qPCR was performed on the Applied Biosystems QuantStudio 5 Real-Time PCR System (Thermo Fisher) with SYBR green qPCR Master Mix (Vazyme) according to the manufacturer’s instructions. The ACE2 mRNA copies were calculated based on a calibration curve obtained by 10-fold stepwise dilution of plasmid DNA. To determine the relative levels of viral RNA (the viral primers valid to detect the genomic RNA or subgenomic RNA) or CACNA1C mRNA in cells, the 2^-ΔΔCT^ method was used [64]. The cycle threshold (CT) of each transcript minus the corresponding CT of the internal control gene 28S rRNA or β-actin was calculated as:

$${\Delta CT}_{sample}={CT}_{sample}-{CT}_{internal\;control}$$

ΔCT _sample_ means ΔCT _experimental sample 1_, ΔCT _experimental sample 2_, ΔCT _experimental sample 3_, ΔCT _control sample 1_, ΔCT _control sample 2_, or ΔCT _control sample 3_.

The relative expression of viral RNA or CACNA1C mRNA relative to control samples was calculated as:

$$Relative\;expression\;(\%)=\frac{2^{-\Delta CT\;sample}}{Average(2^{-\Delta CT\;control\;sample\;1}+2^{-\Delta CT\;control\;sample\;2}+2^{-\Delta CT\;controlsample\;3})}*100\%$$

Experiments were done in three biological replicates. The primer sequences of individual genes are listed in S1 Table.

### Virus infection assay

Cells were infected with the indicated virus in a 100 μL volume for 1 h at 37°C. Then, the cells were washed three times with 2% FBS, and 500 μL of 2% FBS was added to them. Further studies were then carried out.

### Cell viability assay

Cell viability was determined by using the Cell Titer-Glo kit (Promega) following the manufacturer’s instructions. GraphPad Prism software (version 7.0) was used to calculate the CC_50_ concentrations.

### Screening assay

Vero-E6 cells were pretreated with DMSO, diltiazem (1 μM, 10 μM, 100 μM; Sigma), nifedipine (10 μM, 100 μM, 300 μM; Sigma), ethosuximide (10 μM, 100 μM, 300 μM; TOPSCIENCE), or ω-Conotoxin MVIIC (10 nM, 100 nM, 500 nM; APExBIO) at the indicated concentrations for 1 h at 37°C, respectively, then were infected with HRB25 (M.O.I. = 0.01). The inhibitors were present in the culture medium throughout the infection. At 24 h post-infection, the virus titers were determined by use of plaque assays. The IC_50_ of diltiazem was determined as described above.

### Time-of-addition assay

Vero-E6 cells were infected with HRB25 (M.O.I. = 5) for 1 h at 37°C. After the cells were washed, diltiazem (150 μM) was added at -1 h, 1 h, and 2 h post-infection. The virus in the cell lysate was detected by qPCR at 6 h post-infection.

To test whether diltiazem inhibits the late stage of SARS-CoV-2 infection, diltiazem (150 μM) and E64D (100 μM) were added to Vero-E6 cells at 6 h post-infection, and the viral titers in the supernatant were detected by using plaque assays at 24 h post-infection.

### Inhibitor assay

Diltiazem-treated Vero-E6 cells, BEAS-2B cells, or Calu-3 cells were infected with HRB25 at the indicated M.O.I.s. The diltiazem was present throughout the duration of the virus infection. The viral titers in the supernatant or the viral RNA levels in the cell lysate relative to β-actin (Vero-E6 cells) or 28S rRNA (BEAS-2B cells, Calu-3 cells) were measured by using plaque assays or qPCR at 24 h or 48 h post-infection. Vero-E6 were treated with BAPTA-AM (25 μΜ) as described above.

Cells were preincubated with vehicle or diltiazem (150 μΜ) for 1 h at 37°C, then the virus infection assay was performed. Vero-E6 cells were infected with HRB25 (M.O.I. = 5) for 1 h at 37°C. Calu-3 cells were infected with HRB25 (M.O.I. = 10) at 4°C for 1 h. The diltiazem was present throughout the duration of the virus infection. Viral RNA levels in the cell lysate relative to β-actin (Vero-E6 cells) or 28S rRNA (Calu-3 cells) were measured by qPCR at 1 h, 6 h, 12 h, and 24 h post-infection.

Vero-E6 cells were pretreated with diltiazem (150 μM) and remdesivir (10 μM) for 1 h at 37°C, then infected with HRB25 (M.O.I. = 5) for 1 h at 37°C. After the cells were washed, medium contained the indicated inhibitor was added. The virus in the cell lysate was measured by qPCR at 1 h and 6 h post-infection.

HEK293T cells were co-transfected with ACE2-Flag and TMPRSS2-Flag plasmids (HEK293T-ACE2/TMPRSS2 cells) for 48 h. Then, the cells were pretreated with DMSO, camostat mesylate (100 μM), E64D (100 μM), or diltiazem (150 μM) for 1 h at 37°C, and then infected with HRB25 (M.O.I. = 5) for 1 h at 37°C. The inhibitors were present in the culture medium throughout the infection. The viral RNA level in the cell lysate was measured by qPCR at 6 h post-infection. CACNA1C-silenced HEK293T-ACE2/TMPRSS2 cells were treated with the indicated inhibitors as described above.

### Virus neutralization assay

HRB25 (M.O.I. = 5) was mixed with the neutralizing antibody (4A8) (20 μg/mL) or isotype IgG (20 μg/mL) for 1 h at 4°C. Then, Vero-E6 cells were incubated with the mixtures for 1 h at 37°C. The viral RNA level in the cell lysate was measured by qPCR at 1 h and 6 h post-infection.

### Flow cytometry

To detect the expression of ACE2 on cell surface, Vero-E6 cells, Calu-3 cells, A549 cells, HEK293T cells, and Hela cells were seeded onto 6-well plates and harvest by treatment with 0.25% trypsin (without EDTA). The cells were fixed with 3% paraformaldehyde at room temperature for 15 min, then washed three times with FACS wash buffer (PBS containing 2% FCS), and incubated for 2 h with ACE2 antibody (R&D system, AF933) or IgG isotype antibody (Abcam), which served as a control. Cells were then washed and stained with donkey anti-goat Alexa Fluor 488 antibody (Abcam) for 1 h. All cells were analyzed by using an Apogee flow cytometer.

To detect the expression of ACE2 in cells after inhibitor treatment, Vero-E6 cells were treated with diltiazem (150 μM) or BAPTA-AM (25 μM) for 1 h or CACNA1C-silenced for 72 h, then the cells were harvested as described above, and permeabilized with 0.1% saponin (Sigma) for 20 min, before being incubated with an ACE2 antibody to detect the expression of ACE2 as above.

### Viral binding assay

Cells were pretreated with vehicle or diltiazem (150 μΜ) for 1 h at 37°C, then infected with HRB25 (M.O.I. = 10, 1, 0.1, or 0.01) at 4°C for 1 h. Unbound virions were removed by using chilled PBS. Then, the viral RNA level in the cell lysate relative to the β-actin (Vero-E6 cells) or 28S rRNA (Calu-3 cells, Hela cells, A549 cells, and HEK293T cells) was detected by qPCR. The viral binding assay in BAPTA-AM treated cells, or siRNA-transfected cells was then performed as described above.

For the microscopy-based assay of viral binding, diltiazem-treated cells were incubated with HRB25 (M.O.I. = 10) at 4°C for 1 h. After being washed, the cells were fixed, permeabilized with 0.1% Triton X-100 for 15 min, and incubated with 1% BSA. Then the cells were incubated with an anti-SARS-CoV-2 nucleocapsid protein antibody (Sino Biological) overnight at 4°C. After washing, the cells were stained with goat anti-rabbit Alexa Fluor 488 antibody (Thermo Fisher) for 1 h. Nuclei were stained with Hoechst 33342. Image fluorescence intensity was quantified with a Zeiss LSM880 laser-scanning confocal microscope. The resolution of the acquired images was 1024 × 1024. The cell-bound virus fluorescence intensities in at least 110 cells per sample were quantified by using ZEN software.

### Viral internalization assay

Cells were pretreated with vehicle, diltiazem (150 μΜ), or BAPTA-AM (25 μΜ) for 1 h at 37°C, then infected with HRB25 (M.O.I. = 1 or 10) at 4°C for 1 h. After removing the unbound particles by extensive washing, cells were shifted to 37°C for 1 h to initiate internalization. After 1 h, the cells were washed three times for 3 min with acidic buffer (50 mM glycine, 100 mM NaCl, pH 3.0), then 0.25% trypsin was added to remove HRB25 bound to the cell surface. The cells were lysed for total RNA extraction, followed by qPCR to detect internalized viruses. Virus internalization into siRNA-transfected cells was detected as described above.

### Cell-cell fusion assay

The cell-cell fusion assay was performed with HEK293T cells. The target cells and effector cells were respectively seeded in a 5-cm dish at 70%–80% confluence. The target cells were co-transfected with ACE2-VN and TMPRSS2-Flag expression plasmids, whereas the effector cells were co-transfected with SARS-CoV-2 S-VC and pCAGGS-Flag expression plasmids. For the camostat mesylate fusion inhibition assay, camostat mesylate (100 μM) was added into the medium of the effector cells at 6 h after transfection and added to the target cells for 2 h at 24 h post-transfection. For the diltiazem fusion inhibition assay, diltiazem (150 μM) was added into the medium of the effector cells and target cells, respectively, for 2 h at 24 h post-transfection. Then, the effector cells and target cells were washed and resuspended in DMEM containing 10% FBS and the indicated inhibitors, mixed at a 1:1 ratio, and plated in a 24-well plate to incubate at 37°C for 18 h before being imaged under an inverted fluorescence microscope.

### RNAi

siRNA transfections were performed in 24-well plates using the Lipofectamine RNAiMAX transfection reagent (Thermo Fisher Scientific) according to the manufacturer’s instructions. Briefly, siRNA (1 μM, 50 uL per well, Sigma) targeting the CACNA1C or non-targeting siRNA was mixed with 70 μL of OptiMEM medium (Invitrogen) containing 0.8 μL of Lipofectamine RNAiMAX transfection reagent on 24-well plates. After a 30-min incubation at room temperature, cells were seeded into siRNA-coated 24-well plates in a volume of 500 μL per well. HEK293T cells were co-transfected with ACE2-Flag and TMPRSS2-Flag plasmids for 24 h, then RNAi assays were performed as described above. CACNA1C mRNA was assessed by use of qPCR. At 72 h post-transfection, cells were infected with HRB25 for further studies. The siRNA sequences were as follows: siCACNA1C, Sense: 5’- CCUACUUCGUGUCCCUCUUdTdT-3’, Antisense: 5’- AAGAGGGACACGAAGUAGGdTdT-3’.

### Western blot

To detect the expression of ACE2 on the diltiazem-treated or CACNA1C-silenced cell surface, two 10-cm dishes of Vero-E6 cells were used to extract plasma membrane proteins by using the Minute Plasma Membrane Protein Isolation and Cell Fraction Kit (Invent Biotechnologies) following the manufacturer’s instructions. The total ACE2 in cells was extracted with RIPA buffer containing a protease inhibitor. Samples were incubated on ice for 30 min and centrifuged at 12,000 × *g* for 20 min at 4°C. Clarified cell lysate was diluted in denaturing SDS gel loading buffer and boiled for 15 min.

The samples were loaded onto a 4%–12% SDS-PAGE gel (Genscript) and separated by electrophoresis. Proteins were transferred to a polyvinylidene difluoride (PVDF) membrane (Merck-Millipore). The PVDF membrane was blocked with 5% skim milk and incubated with anti-Flag antibody (Genscript), anti-Myc antibody (Genscript), anti-ACE2 antibody (R&D system), anti-β-actin antibody (Zsbio), and anti-Zonula occludens protein 3 (ZO3) antibody (abcam). After being washed, the PVDF membrane was incubated with the following HRP-conjugated secondary antibodies: goat anti-rabbit HRP (Genscript), goat anti-mouse HRP (Genscript), and rabbit anti-goat HRP (Jackson ImmunoResearch). Signals were detected by using the enhanced chemiluminescence (ECL) reagent (Merck Millipore).

### Co-immunoprecipitation

HEK293 cells were transfected with plasmids by using TransIT-293 transfection reagent following the manufacturer’s instructions. At 48 h post-transfection, the cells were lysed with 1% NP-40 PBS buffer for 1 h at 4°C. Supernatant was collected and mixed with 40 μL of protein G agarose (Roche) for 4 h at 4°C to remove non-specific binding proteins in the supernatant. After being washed, the supernatant was mixed with anti-Flag antibody-conjugated agarose beads (Sigma) for 6 h at 4°C. The beads were isolated by centrifugation, washed five times with 1% NP-40 PBS buffer, and used for western blotting.

### Pull-down assay

The soluble SARS-CoV-2 S1 protein (SARS-CoV-2 S1-His, aa 14–681) was expressed in transfected BHK-21 cells and purified for the pull-down assay. The whole cell lysate from HEK293 cells expressing Ca_v_1.2 α_1c_-Flag proteins was mixed with Protein G Agarose for 4 h at 4°C. Then the supernatant was mixed with anti-Flag antibody-conjugated agarose beads for 4 h at 4°C. After conjugation, the beads were washed three times with 1% NP-40 PBS buffer, Then, the soluble SARS-CoV-2 S1-His protein (10 μg) was mixed with beads at 4°C for 5 h on a flip shaker and washed five times with 1% NP-40 PBS buffer. After the final wash, the samples were subjected to SDS-PAGE, and assessed by western blot analysis.

### Confocal immunofluorescence microscopy

Vero-E6 cells were transfected with Ca_v_1.2 α_1c_-Flag. At 24 h after transfection, cells were inoculated with HRB25 (M.O.I. = 10) for 1 h on ice, and unbound virions were removed and fixed in 4% paraformaldehyde for 15 min. Multiplex immunofluorescence with Tyramide Signal Amplification was performed by following the previously established protocol [65]. The primary antibodies used in this study were anti-Flag (Sigma), anti-ACE2 (Abcam) and anti-SARS-CoV-2 nucleocapsid protein (Sino Biological). The secondary antibodies were HRP-conjugated anti-rabbit IgG (Zsbio) and HRP-conjugated anti-mouse IgG (Zsbio). Images were acquired using a Zeiss LSM880 laser-scanning confocal microscope equipped with Airyscan. Cells were scanned 24 layers along the Z axis with a pixel dwell time of 1 microsecond. The resolution of the acquired images was 2048 × 2048.

To quantify the colocalization of SARS-CoV-2, ACE2, and Ca_v_1.2 α_1c_-Flag, data from single channels were processed using the “surface module” of Bitplane Imaris software (Bitplane AG, Zurich, Switzerland), and then merged the produce images to observe the colocalization of SARS-CoV-2, ACE2, and Ca_v_1.2 α_1c_-Flag. The 3D-rendered images were generated by using Imaris software.

### Animal experiments

Six-week-old female BALB/c mice were lightly anesthetized with CO_2_ and treated intramuscularly (5 mg/kg) or intranasally (0.01 mg/kg) with diltiazem, followed by a daily maintenance dose of 5 mg/kg or 0.01 mg/kg. As a control, 6-week-old female BALB/c mice were administrated vehicle (water) daily. At 1 h after administration of the loading dose of diltiazem or vehicle, the mice were inoculated intranasally with 30 PFU of HRB26M in a volume of 50 μL. To test the therapeutic effect of diltiazem after SARS-CoV-2 infection, HRB26M-infected mice were intramuscularly treated with diltiazem (5 mg/kg) at 6 h post-infection, followed by a daily maintenance dose of 5 mg/kg. On day 3 post-inoculation, the mice were euthanized and their lungs were collected for qPCR virus detection, plaque assays, or immunohistochemical assays.

To examine the protective effects of diltiazem in the lethal mouse model, human ACE2 transgenic C57BL/6J mice (K18-hACE2) were intramuscularly inoculated with diltiazem (5 mg/kg) or vehicle. One hour later, the mice were intranasally infected with HRB25 at a dose of 100 PFU, followed by a daily maintenance dose of 5 mg/kg diltiazem administered for 2 days. The mice were housed and observed for 12 days to monitor survival and body weight changes.

### Immunohistochemical (IHC) assay

The immunohistochemical assay was performed as previously described [66]. Primary rabbit anti-SARS-CoV-2 nucleocapsid protein monoclonal antibody (Frdbio) and HRP-conjugated anti-rabbit IgG secondary antibody (Sigma) were used. Immunostaining was visualized with DAB and counterstained with hematoxylin.

### Statistical analysis

Quantitative data are presented as means ± standard deviations (SDs) of three independent experiments or replicates. The statistical analysis of the normalized data was performed in Microsoft Excel using an unpaired two-tailed Student’s t-test. The statistical details are given in the figure legend. Significance levels were as follows: ns, not significant, *p < 0.05, **p < 0.01, ***p < 0.001.

## Supporting information

S1 Fig BAPTA-AM has no effect on cell binding or internalization of SARS-CoV-2. (A and B) Vero-E6 cells (A) and A549 cells (B) were treated with diltiazem or BAPTA-AM for 1 h, and then incubated with HRB25 (M.O.I. = 10) at 4°C for 1 h. The viral RNA level in the cell lysate was measured by qPCR. (C) BAPTA-AM-treated Vero-E6 cells were infected with HRB25 (M.O.I. = 0.01), the supernatants were harvested at 24 h post-infection for plaque assays. (D and E) Vero-E6-ACE2 cells were treated with BAPTA-AM for 1 h, and then incubated with HRB25 (M.O.I. = 10) at 4°C for 1 h and washed with PBS, then shifted to 37°C for 1 h. The cells were then washed with PBS (D) or acid buffer/trypsin (E). The washed cells were lysed for qPCR to detect SARS-CoV-2 binding to cells (D) or internalized into cells (E). The data shown are the means ± SDs of three independent experiments or replicates. The two-tailed unpaired Student’s t-test was used for the statistical analysis. ns, not significant, **p < 0.01, ***p < 0.001.
(TIF)

S2 Fig BAPTA-AM has no effect on the cell surface expression of ACE2. Vero-E6 cells were treated with BAPTA-AM for 1 h, and then the expression of ACE2 on the cell surface and in total cells was detected by use of flow cytometry. The data shown are representative of three independent experiments.
(TIF)

S3 Fig Different doses of diltiazem inhibit the internalization of SARS-CoV-2. Vero-E6 cells were incubated with vehicle or diltiazem at the indicated concentrations for 1 h, and then incubated with HRB25 (M.O.I. = 10) at 4°C for 1 h and washed with PBS, then shifted to 37°C for 1 h. The cells were then washed with acid buffer/trypsin. The viral RNA levels in the cell lysates were detected by qPCR. The data shown are the means ± SDs of three independent experiments. The two-tailed unpaired Student’s t-test was used for the statistical analysis. **p < 0.01.
(TIF)

S1 Table List of primers for qPCR used in this study. (DOCX)
