# Supplementary figures and images for: Diltiazem inhibits SARS-CoV-2 cell attachment and internalization and decreases the viral infection in mouse lung
Source: PLoS Pathog. 2022 Feb 17;18(2):e1010343. doi: 10.1371/journal.ppat.1010343 (PMC8890723; doi:10.1371/journal.ppat.1010343)

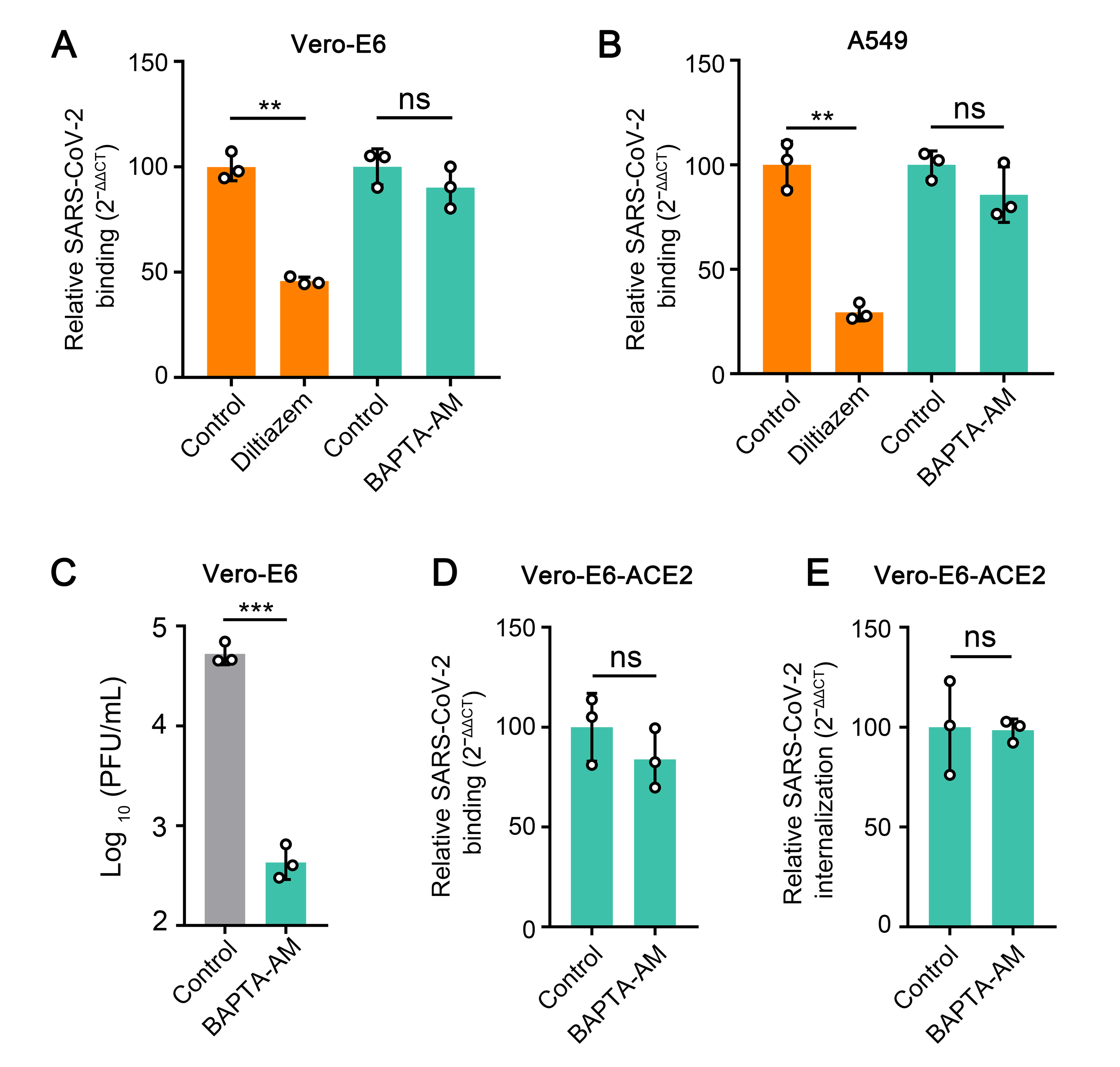

Supplement: S1 Fig — (A and B) Vero-E6 cells (A) and A549 cells (B) were treated with diltiazem or BAPTA-AM for 1 h, and then incubated with HRB25 (M.O.I. = 10) at 4°C for 1 h. The viral RNA level in the cell lysate was measured by qPCR. (C) BAPTA-AM-treated Vero-E6 cells were infected with HRB25 (M.O.I. = 0.01), the supernatants were harvested at 24 h post-infection for plaque assays. (D and E) Vero-E6-ACE2 cells were treated with BAPTA-AM for 1 h, and then incubated with HRB25 (M.O.I. = 10) at 4°C for 1 h and washed with PBS, then shifted to 37°C for 1 h. The cells were then washed with PBS (D) or acid buffer/trypsin (E). The washed cells were lysed for qPCR to detect SARS-CoV-2 binding to cells (D) or internalized into cells (E). The data shown are the means ± SDs of three independent experiments or replicates. The two-tailed unpaired Student’s t-test was used for the statistical analysis. ns, not significant, **p < 0.01, ***p < 0.001. (TIF) [file ppat.1010343.s001.tif]

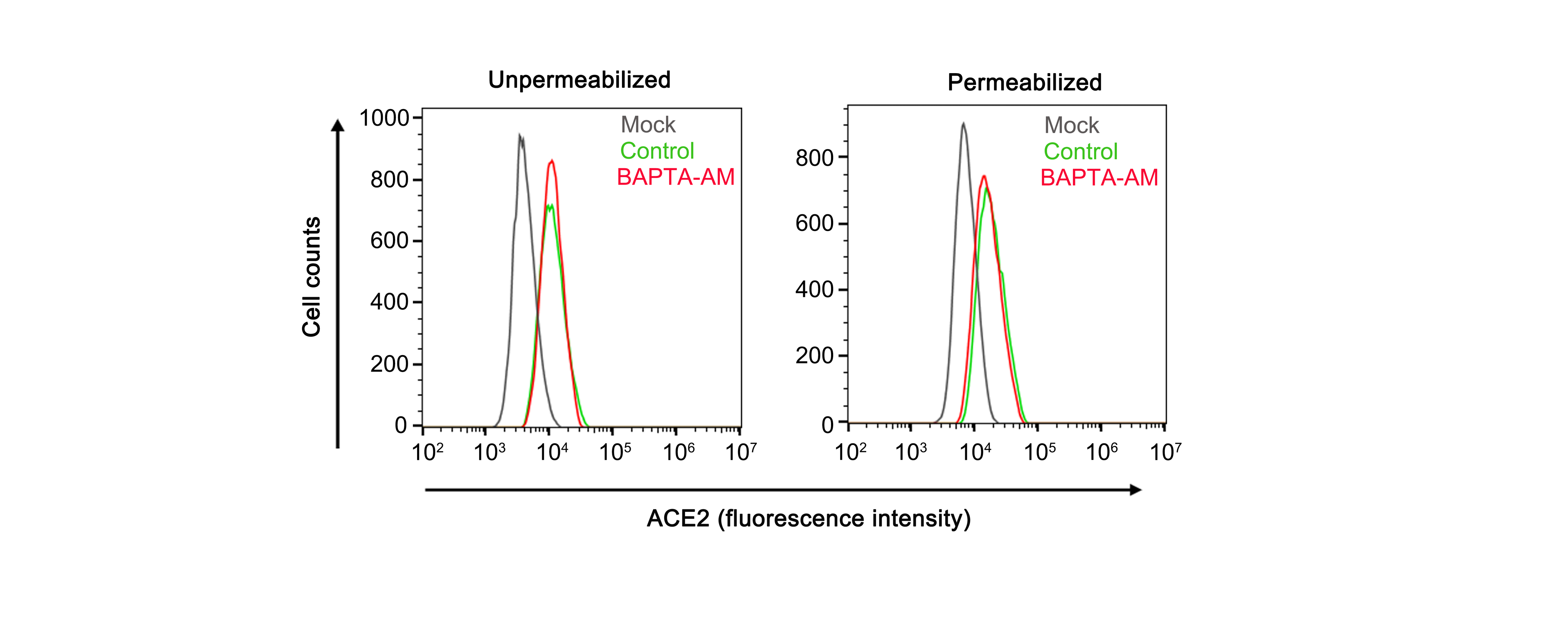

Supplement: S2 Fig — Vero-E6 cells were treated with BAPTA-AM for 1 h, and then the expression of ACE2 on the cell surface and in total cells was detected by use of flow cytometry. The data shown are representative of three independent experiments. (TIF) [file ppat.1010343.s002.tif]

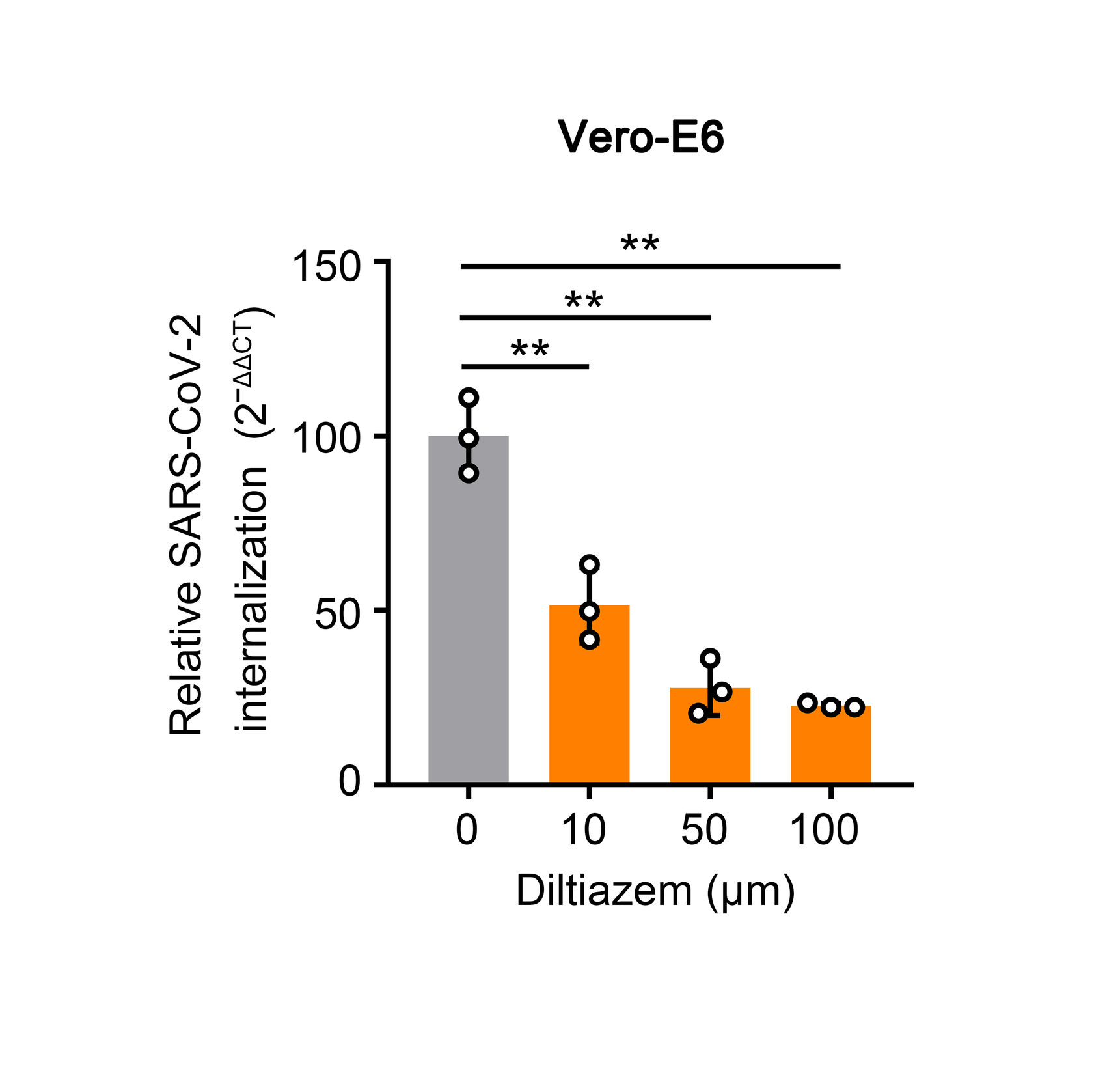

Supplement: S3 Fig — Vero-E6 cells were incubated with vehicle or diltiazem at the indicated concentrations for 1 h, and then incubated with HRB25 (M.O.I. = 10) at 4°C for 1 h and washed with PBS, then shifted to 37°C for 1 h. The cells were then washed with acid buffer/trypsin. The viral RNA levels in the cell lysates were detected by qPCR. The data shown are the means ± SDs of three independent experiments. The two-tailed unpaired Student’s t-test was used for the statistical analysis. **p < 0.01. (TIF) [file ppat.1010343.s003.tif]
