# Supplementary material for: Diltiazem inhibits SARS-CoV-2 cell attachment and internalization and decreases the viral infection in mouse lung
Source: PLoS Pathog. 2022 Feb 17;18(2):e1010343. doi: 10.1371/journal.ppat.1010343 (PMC8890723; doi:10.1371/journal.ppat.1010343)
Supplement: S1 Table — (DOCX) [file ppat.1010343.s004.docx]

**S1 Table. List of primer for qPCR used in this study.**

| **Primers** | **Sequence (5’→3’)** |  |
| --- | --- | --- |
| CACNA1C-human-qPCR-F  CACNA1C-human-qPCR-R  CACNA1C-monkey-qPCR-F  CACNA1C-monkey-qPCR-R  β-actin-monkey-qPCR-F  β-actin-monkey-qPCR-R  SARS-CoV-2-N-qPCR-F  SARS-CoV-2-N-qPCR-R  28s rRNA-human-qPCR-F  28s rRNA-human-qPCR-R  ACE2-qPCR-F  ACE2-qPCR-R | GTCACCTTTCAGGAGCAGGG  AGGTGGAGTTGACCACGTACC  GAGGAAGAGGAGAAGGAGAGAA  TCAGCCGTGATGGATTTCAG  GACAGGATGCAGAAGGAGATTAC  CTGCTTGCTGATCCACATCT  GGGGAACTTCTCCTGCTAGAAT  CAGACATTTTGCTCTCAAGCTG  GGGTGGTAAACTCCATCTAAGG  GCCCTCTTGAACTCTCTCTTC  TGGGACTCTGCCATTTACTTAC  CCCAACTATCTCTCGCTTCATC |  |
